# Supplementary material for: Structural Basis for Specific Interaction of TGFβ Signaling Regulators SARA/Endofin with HD-PTP
Source: Structure. 2017 Jul 5;25(7):1011–1024.e4. doi: 10.1016/j.str.2017.05.005 (PMC5501724; doi:10.1016/j.str.2017.05.005)
Supplement: Document S1. Figures S1–S3 and Table S1 [file mmc1.pdf]

Structure, Volume *25*

Supplemental Information

Structural Basis for Specific Interaction of TGFβ Signaling Regulators SARA/Endofin with HD-PTP

Deepankar Gahloth, Colin Levy, Louise Walker, Lydia Wunderley, A. Paul Mould, Sandra Taylor, Philip Woodman, and Lydia Tabernero

Supplementary Information

Supplementary Figures S1, S2, S3; Supplementary Table 1

Figure S1 (related to Figure 1 and Figure 2)

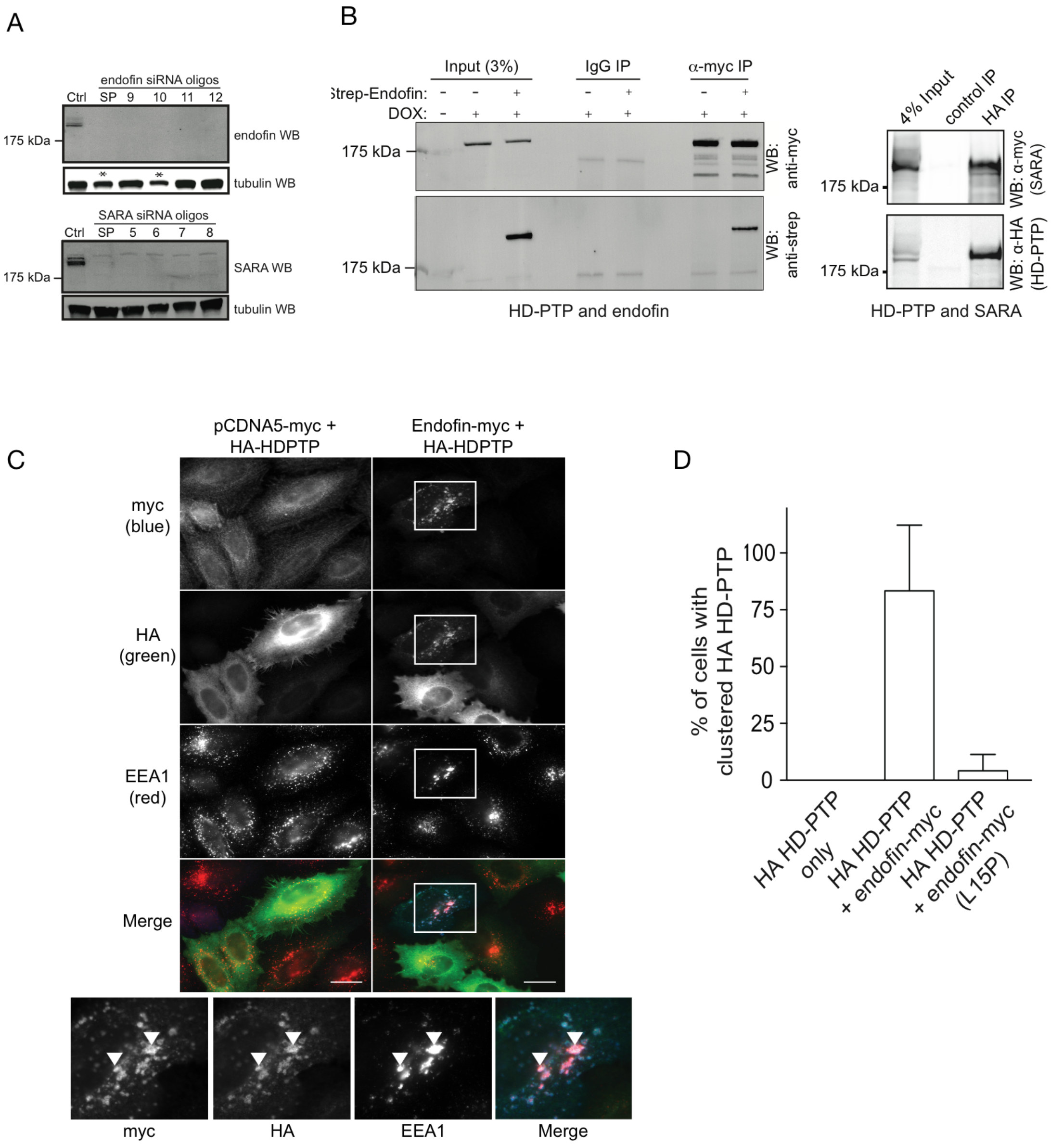

(A) HeLaM cells were transfected with siRNAs against endofin, SARA, or with a control siRNA and extracts were immunoblotted for endofin or SARA, with tubulin as a loading control. Note that endofin oligo 10 was mildly toxic (asterisks). (B) Left panel: HD-PTP-myc Flip-In HeLa cells were transiently

transfected with endofin-Strep-tag and induced with doxycycline (DOX). Control IgG or anti-myc IPs were blotted as indicated. Right panel: HEK293 cells co-expressing HA-HD-PTP and SARA-myc were subjected to control or anti-HA IPs and immunoblotted as indicated. (C) Cells were transfected with empty vector and HA-HD-PTP (left panels), or with endofin-myc and HA-HD-PTP (right panels), and stained with anti-myc (blue) and anti-HA (green), as well as with anti-EEA1 (red) to label early endosomes. Bars = 10 µm. Boxed regions are magnified x 3 and displayed below. Arrowheads indicate examples of co-localisation between HA-HD-PTP and endofin-myc occurring on early endosomes. (D) Cells were transfected with HA-HD-PTP and WT endofin-myc or endofin-myc^{L15P}. Colocalisation was scored in 3 independent experiments. Values are means +/- SD.

Figure S2 (related to figure 4)

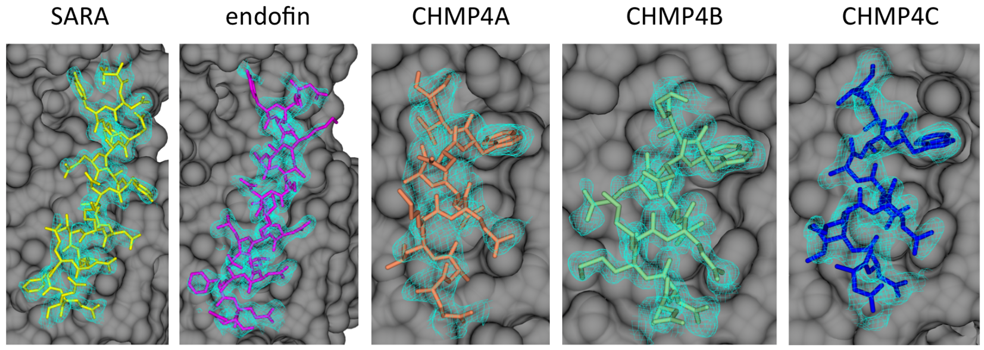

Electron density maps for the peptides in each structure presented in the study. Each of the five peptides (SARA - yellow, endofin - purple, CHMP4A - orange, CHMP4B - green & CHMP4C - blue) is shown in stick representation along with a surface rendering of their respective HD-PTP domains (grey). Fo-Fc omit electron density (cyan) contoured at 2 sigma is shown for each

peptide. Figure generated in CCP4MG v2.10.4 (McNicholas et al., 2011).

Figure S3 (related to Figure 7)

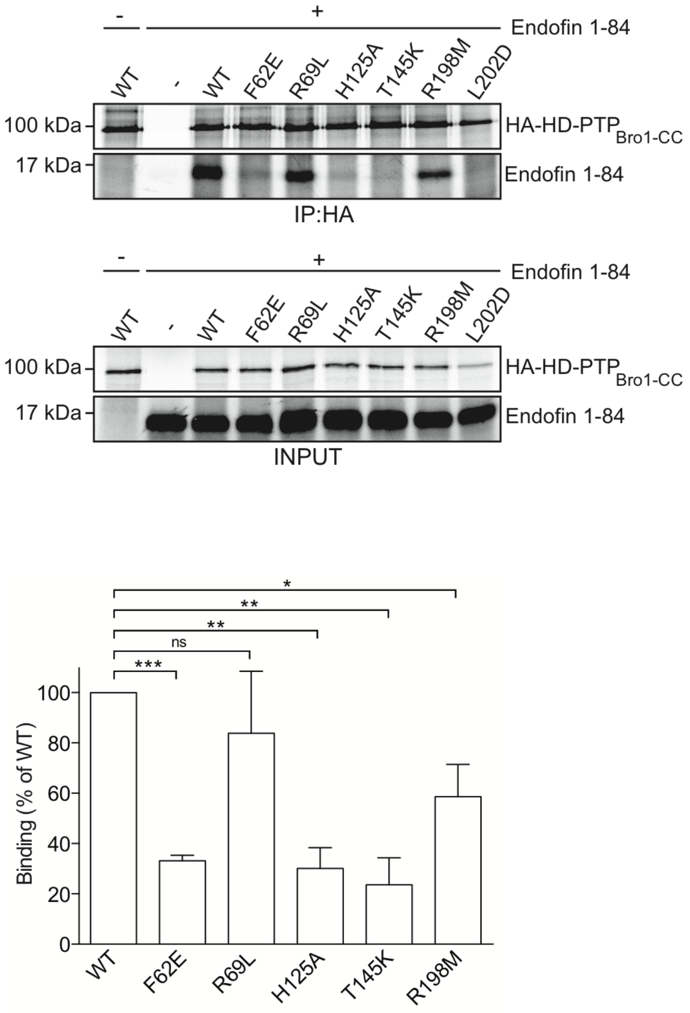

Top: wild type (WT) HA-HD-PTP$_{Bro1-CC}$ or the indicated mutants were co-translated with endofin1-84-myc in reticulocyte lysates. Translation products (input) are displayed in the lower panels. Samples were immunoprecipitated with anti-HA beads and examined by phosphorimaging to identify HA-HD-PTP$_{Bro1-CC}$ and co-immunoprecipitated endofin1-84-myc (upper panels). Bottom: data from 3 independent experiments +/- SD. Note that data for the L202D mutant were not included in the quantitation because this mutant

translated poorly compared to the other constructs. Mutation of HA-HD-$PTP_{Bro1-CC}$ residues F62, H125 and T145 at the S-site and of L202 at the common site impair binding of the endofin 1-84 fragment. Mutation of R69 or R198 had a milder effect with R69 not significantly different from WT. Statistics were performed using the unpaired t-test with Welch's correction: ***: $p<0.001$; **: $p<0.01$; *: $p<0.05$.

Table S1. HD-PTP interactors revealed in a Y2H screen (related to STAR Methods section).

HD-PTP$_{Bro1\text{-}CC}$ was used as bait to screen a placental cDNA library for interactors. The following ESCRT and endocytic proteins were identified (number of clones shown in brackets). Binding to UBAP1 and STAM2 have already been reported (Stefani et al., 2011, Ali et al., 2013).

ESCRTs/ESCRT-related	Endosomal
CHMP4B (1)	Endofin (5)
CHMP5 (1)	SARA (4)
STAM2 (5)	Rabep1 (7)
UBAP1 (5)	
SPG20 (3)	
