# Supplementary material for: Structural Basis for Specific Interaction of TGFβ Signaling Regulators SARA/Endofin with HD-PTP
Source: Structure. 2017 Jul 5;25(7):1011–1024.e4. doi: 10.1016/j.str.2017.05.005 (PMC5501724; doi:10.1016/j.str.2017.05.005)
Supplement: Document S2. Article plus Supplemental Information [file mmc2.pdf]

Structure

Article

Structural Basis for Specific Interaction of TGFβ Signaling Regulators SARA/Endofin with HD-PTP

Graphical Abstract

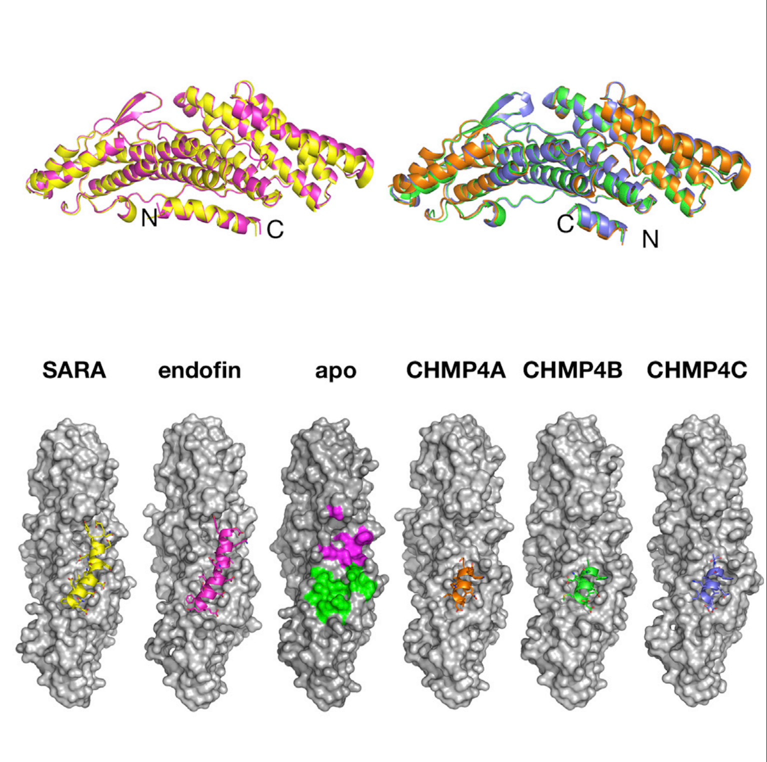

Highlights

- SARA and endofin interact with HD-PTP phosphatase with high affinity
- Structure of HD-PTP_{Bro1} with SARA, endofin, and CHMP4 shows a common binding site
- SARA/endofin exploit a unique specificity pocket in HD-PTP

Authors

Deepankar Gahloth, Colin Levy, Louise Walker, ..., Sandra Taylor, Philip Woodman, Lydia Tabernero

Correspondence

philip.woodman@manchester.ac.uk (P.W.),
lydia.tabernero@manchester.ac.uk (L.T.)

In Brief

Gahloth et al. report structures of HD-PTP_{Bro1} with SARA, endofin, and CHMP4. The structures reveal a common binding site, but specific interactions of SARA/endofin at a unique pocket in HD-PTP rationalizes their selective and high-affinity binding and provides new insight into the endocytic regulation of TGFβ/BMP signaling.

Gahloth et al., 2017, Structure *25*, 1011–1024
July 5, 2017 © 2017 The Authors. Published by Elsevier Ltd.
http://dx.doi.org/10.1016/j.str.2017.05.005

CellPress

Structure
Article

Structural Basis for Specific Interaction of TGFβ Signaling Regulators SARA/Endofin with HD-PTP

Deepankar Gahloth,[1,2] Colin Levy,[1,2] Louise Walker,[1,2] Lydia Wunderley,[1,2] A. Paul Mould,[1] Sandra Taylor,[1] Philip Woodman,[1,*] and Lydia Tabernero[1,3,*]
[1]School of Biological Sciences, Faculty of Biology Medicine and Health, University of Manchester, Manchester Academic Health Science Centre, Manchester M13 9PT, UK
[2]These authors contributed equally
[3]Lead Contact
*Correspondence: philip.woodman@manchester.ac.uk (P.W.), lydia.tabernero@manchester.ac.uk (L.T.)
http://dx.doi.org/10.1016/j.str.2017.05.005

SUMMARY

SARA and endofin are endosomal adaptor proteins that drive Smad phosphorylation by ligand-activated transforming growth factor β/bone morphogenetic protein (TGFβ/BMP) receptors. We show in this study that SARA and endofin also recruit the tumor supressor HD-PTP, a master regulator of endosomal sorting and ESCRT-dependent receptor downregulation. High-affinity interactions occur between the SARA/endofin N termini, and the conserved hydrophobic region in the HD-PTP Bro1 domain that binds CHMP4/ESCRT-III. CHMP4 engagement is a universal feature of Bro1 proteins, but SARA/endofin binding is specific to HD-PTP. Crystallographic structures of HD-PTP$_{Bro1}$ in complex with SARA, endofin, and three CHMP4 isoforms revealed that all ligands bind similarly to the conserved site but, critically, only SARA/endofin interact at a neighboring pocket unique to HD-PTP. The structures, together with mutagenesis and binding analysis, explain the high affinity and specific binding of SARA/endofin, and why they compete so effectively with CHMP4. Our data invoke models for how endocytic regulation of TGFβ/BMP signaling is controlled.

INTRODUCTION

Endosomes are hubs for regulating cell surface receptor-dependent signaling pathways (Mellman and Yarden, 2013; von Zastrow and Sorkin, 2007). Signaling is maintained if internalized activated receptors remain resident in the endosome or are recycled to the cell surface. Alternatively, signaling is downregulated if receptors are targeted to lysosomes for degradation. Lysosomal delivery first requires sorting of receptors to intraluminal vesicles (ILVs) within the multivesicular body (MVB), a process that is orchestrated by ESCRT (Endosomal Sorting Complex Required for Transport) complexes (ESCRT-0 to -III) (Hurley, 2015; Olmos and Carlton, 2016; Schuh and Audhya, 2014). The ESCRT pathway is critical for downregulating EGFR (epidermal growth factor receptor) (Eden et al., 2012), PDGFR (platelet-derived growth factor receptor) (Ma et al., 2015), VEGFR (vascular endothelial growth factor receptor) (Lampugnani et al., 2006), Notch (Vaccari and Bilder, 2005), TLR (Toll-like receptor) (Husebye et al., 2006), and GPCR (G-protein-coupled receptor) (Marchese et al., 2003). Likewise, ESCRT-mediated degradation of E-cadherin (Palacios et al., 2005) and α5β1 integrin (Kharitidi et al., 2015; Lobert et al., 2010) controls cell adhesion and migration. Crucial for these processes is the assembly of ESCRT-III filaments that drive invagination of the endosomal membrane during ILV formation (Schoneberg et al., 2016).

The endosomal pathway also plays an essential role in regulating transforming growth factor β/bone morphogenetic protein (TGFβ/BMP) signaling (Chen, 2009; Di Guglielmo et al., 2003; Felberbaum-Corti et al., 2003; Hata and Chen, 2016). Activated TGFβ/BMP receptors are internalized and form SARA/endofin-Smad complexes at the early endosome (Chen et al., 2007; Shi et al., 2007; Shi and Massague, 2003). This results in phosphorylation of Smad proteins, which then form complexes with Smad4 and translocate to the nucleus to regulate gene expression (Hayes et al., 2002; Panopoulou et al., 2002). An alternative internalization pathway drives the degradation of TGFβ/BMP receptors, via Smurf and Smad6/7, thus turning off downstream signaling (Di Guglielmo et al., 2003; Imamura et al., 2013; Kavsak et al., 2000; Zuo et al., 2013). Hence, a balance between receptor internalization, recycling, and degradation is key for controlling TGFβ/BMP signaling responses (Chen, 2009; Di Guglielmo et al., 2003; Ehrlich, 2016; Hata and Chen, 2016; Heldin and Moustakas, 2016).

SARA (smad anchor for receptor activation) and endofin (endosome-associated FYVE domain protein) are key positive regulators of TGFβ and BMP signaling, respectively. SARA is an essential component of Smad-dependent signaling, where it recruits Smad2/Smad3 to TGFβ-activated receptors (Tsukazaki et al., 1998). Endofin acts as the Smad1/5/8 anchor to BMP-activated receptors (Goh et al., 2015; Shi et al., 2007). Endofin can also function in the TGFβ pathway (Chen et al., 2007) by interacting with several type-I TGFβ receptors. SARA and endofin each contain an FYVE (Fab1 YOTB Vac1 EEA1) domain (Figure 1A) that localizes them to the early endosome (Panopoulou et al., 2002; Seet and Hong, 2001; Tsukazaki et al., 1998).

His domain protein tyrosine phosphatase (HD-PTP/PTPN23) is a tumor suppressor that regulates mitogenic receptor downregulation, endocytic recycling, and cell migration (Chen et al., 2012; Doyotte et al., 2008; Kharitidi et al., 2015; Lee et al.,

 © 2017 The Authors. Published by Elsevier Ltd.
This is an open access article under the CC BY license (http://creativecommons.org/licenses/by/4.0/).

CellPress

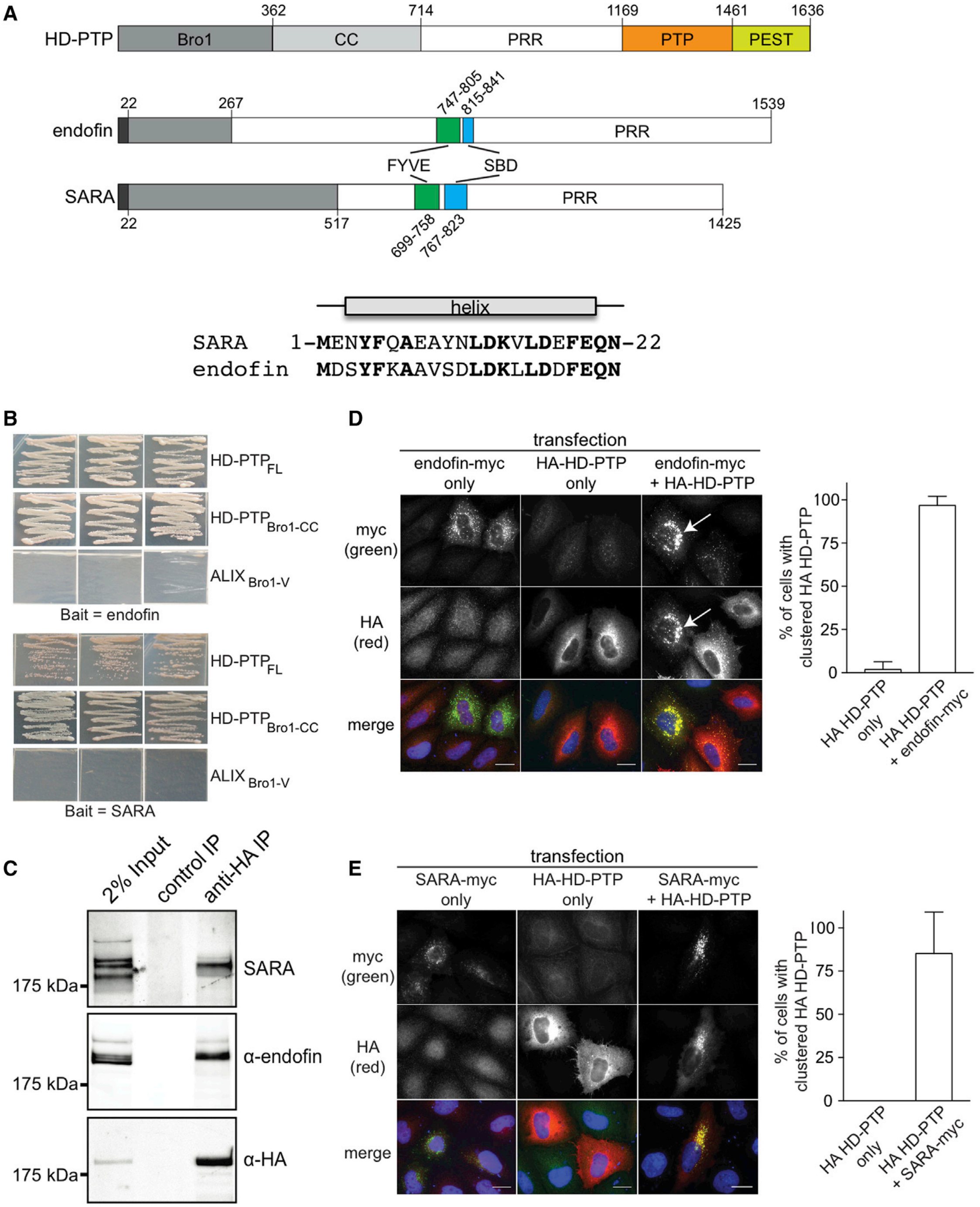

Figure 1. HD-PTP Binds to the Smad Regulators SARA and Endofin at the Endosome

(A) Top: domain organization of HD-PTP (CC, coiled-coil domain; PRR, proline-rich region; PTP, protein tyrosine phosphatase domain). Bottom: domain organization of endofin and SARA (FYVE, Fab1, YOTB, Vac1, and EEA1 zinc-finger domain; SBD, Smad binding domain; PRR, proline-rich region). In gray are the minimal HD-PTP interacting regions on endofin and SARA identified by the Y2H screen; black indicates the N-terminal 22-residue regions predicted to form α helices, with sequences shown below and identical residues highlighted in bold.

(B) Y2H assays show that endofin and SARA interact with HD-PTP$_{FL}$ (full-length) or HD-PTP$_{Bro1\text{-}CC}$, but not with Alix$_{Bro1\text{-}V}$. Yeast cells were transformed in triplicate with the identified prey constructs and full-length endofin or SARA bait constructs, and selected for interactions on QDO plates.

(C) HA-HD-PTP co-immunoprecipitates endogenous endofin and SARA from cell lysates.

(D and E) Co-expression of endofin-myc (D) or SARA-myc (E) redistributes HA-HD-PTP onto enlarged myc-labeled endosomes (indicated by arrows). Left panels are example images; right panels are quantitation from three independent experiments (mean ± SD). Scale bars, 10 μm.

2007; Ma et al., 2015; Manteghi et al., 2016). Specifically, HD-PTP is a key regulator of ESCRT-dependent sorting of cell surface receptors such as activated EGFR (Doyotte et al., 2008), PDGFR (Ma et al., 2015), and integrins (Kharitidi et al., 2015), and drives their degradation by coordinating trafficking to the MVB. Specifically, HD-PTP cooperates with multiple ESCRTs to help sequester ubiquitinated cargo away from recycling pathways and drive its incorporation into ILVs within the developing MVB (Ali et al., 2013; Doyotte et al., 2008; Stefani et al., 2011). A central feature of HD-PTP function is the ability of its Bro1 domain to recruit CHMP4, the major subunit of the ESCRT-III membrane remodeling protein complex (Doyotte et al., 2008; Ichioka et al., 2007; Parkinson et al., 2015).

Here we report that HD-PTP also associates with SARA and endofin at the early endosome. These are high-affinity interactions that overlap with the conserved CHMP4 binding site in the Bro1 domain. However, the binding of SARA and endofin is specific to HD-PTP, in contrast to that of CHMP4, which binds multiple Bro1 proteins including Alix and Brox (Ichioka et al., 2007, 2008). Crystallographic structures of the HD-PTP$_{Bro1}$ domain in complex with the relevant binding regions of SARA, endofin, and all three human CHMP4 isoforms reveal that SARA and endofin binding is similar to that of CHMP4 at the conserved site, but critically extends into a neighboring specific pocket unique to HD-PTP. Altogether, this study identifies the Bro1 domain of HD-PTP as a highly selective interaction hub that coordinates recruitment of endosomal signaling adaptors versus MVB sorting and receptor degradation.

RESULTS

The TGFβ/BMP-Dependent SMAD Signaling Regulators SARA and Endofin Bind to HD-PTP at the Endosome

HD-PTP is a multidomain protein (Figure 1A). Its minimal functional region includes the first two domains (Doyotte et al., 2008); Bro1 and the coiled coil (CC) that adopts an extended conformation (Gahloth et al., 2016). An HD-PTP$_{Bro1\text{-}CC}$ construct was used as bait in a yeast two-hybrid (Y2H) screen to identify new interacting partners of HD-PTP that could be relevant to ESCRT function on the endocytic pathway. This screen identified ESCRT components, with several endosomal proteins also yielding multiple clones (Table S1). Notable were the endocytic Smad adaptors, SARA and endofin, which we focus on in this study. Directed Y2H confirmed that both proteins interact with HD-PTP$_{Bro1\text{-}CC}$, as well as with the full-length protein (HD-PTP$_{FL}$) (Figure 1B). These interactions are selective for HD-PTP$_{Bro1\text{-}CC}$, since neither endofin nor SARA interacted with the analogous Bro1-V region from the related protein Alix (Figure 1B). Interaction of HD-PTP with both SARA and endofin in cells was then confirmed by co-immunoprecipitation (coIP) of endogenous SARA and endofin with hemagglutinin (HA)-tagged HD-PTP (Figure 1C) (the SARA and endofin antibodies were validated using small interfering RNA [siRNA]; Figure S1A). Furthermore, interactions were observed between co-expressed HD-PTP and endofin/SARA (Figure S1B).

Endofin and SARA both localize to early endosomes via their FYVE domains (Seet and Hong, 2001). Thus we tested whether they can interact with HD-PTP at this location. We found that HA-HD-PTP was predominantly cytosolic when expressed alone (Figures 1D and 1E). In contrast, when co-expressed with endofin-myc (Figure 1D) or SARA-myc (Figure 1E), HA-HD-PTP distributed strongly to clusters that labeled with anti-myc or with the early endosomal marker, EEA1 (Figure S1C). Hence, HD-PTP forms novel interactions with these two Smad-signaling regulator proteins, SARA and endofin, and this association results in recruitment of expressed HD-PTP to the early endosome.

The N-Terminal Regions of SARA and Endofin Interact with the Bro1 Domain of HD-PTP

The minimal region in endofin that interacted with HD-PTP was mapped to the first 84 residues by Y2H (Figure 2A). Binding of HD-PTP to the endofin N terminus was confirmed by coIP of in vitro translated endofin(1–84) with HD-PTP$_{Bro1\text{-}CC}$ (Figure 2B). This N-terminal region is highly conserved between SARA and endofin, and the first 22 residues are predicted (using PsiPred; http://bioinf.cs.ucl.ac.uk/psipred/) to form an α helix (Figure 1A). Furthermore, mutation of a core leucine residue (L15) to proline is predicted to disrupt this helix. Indeed, mutation of L15 to P (or to a charged residue; E) prevented binding of endofin to HD-PTP$_{Bro1\text{-}CC}$, whereas mutation to its cognate valine in SARA (L15 to V) did not affect binding (Figures 2A and 2B). Finally, endofin^{L15P} was unable to induce the endosomal clustering of co-expressed HD-PTP (Figure S1D), confirming that the N-terminal helix is important for binding to HD-PTP within cells.

We then demonstrated by biosensor binding experiments that the first 22 residues of endofin, bound to HD-PTP$_{Bro1}$ and HD-PTP$_{Bro1\text{-}CC}$ with similar high affinity (dissociation constant K_D 3.1 ± 0.1 μM and 2.7 ± 0.1 μM, respectively), suggesting that the endofin binding site is in the Bro1 domain of HD-PTP (Figure 2C). The endofin 22-mer peptide did not bind to the Bro1 domain of Alix (Figure 2D), confirming the binding selectivity indicated by Y2H (Figure 1B). Furthermore, an endofin peptide containing the L15P mutation did not bind to HD-PTP$_{Bro1}$ or HD-PTP$_{Bro1\text{-}CC}$ (Figures 2E and 2F), confirming that the integrity of the N-terminal helix is important for interaction. We also showed that the equivalent region of SARA binds to HD-PTP$_{Bro1}$ (K_D 11.3 ± 0.3 μM) (Figure 2C). Altogether, these findings identify N-terminal helices within endofin and SARA as important for binding to the HD-PTP Bro1 domain.

Endofin Competes with CHMP4 for Binding to HD-PTP$_{Bro1}$

The Bro1 domain of HD-PTP contains a conserved hydrophobic site, also present in other Bro1 proteins including Alix and Brox (McCullough et al., 2008; Mu et al., 2012), for interaction with the ESCRT-III subunit CHMP4 (charged multivesicular body protein 4, of which there are three isoforms; CHMP4A, B, and C). We have previously reported that the HD-PTP hydrophobic residues L202/I206 are critical for CHMP4 interaction, since the double mutation L202D/I206D completely blocked binding to CHMP4 in cell extracts while the single L202D mutation substantially impaired binding (Doyotte et al., 2008). Here we show that the double L202D/I206D mutation also prevented the recruitment of HD-PTP to the endosome when co-expressed with endofin-myc (Figure 3A; the mutant localized with endofin-myc in 0% of cells, whereas wild-type HA-HD-PTP localized with endofin-myc in 100% of cells, from two independent experiments). In addition, the endofin peptide did not bind to the HD-PTP$_{Bro1\text{-}CC}$

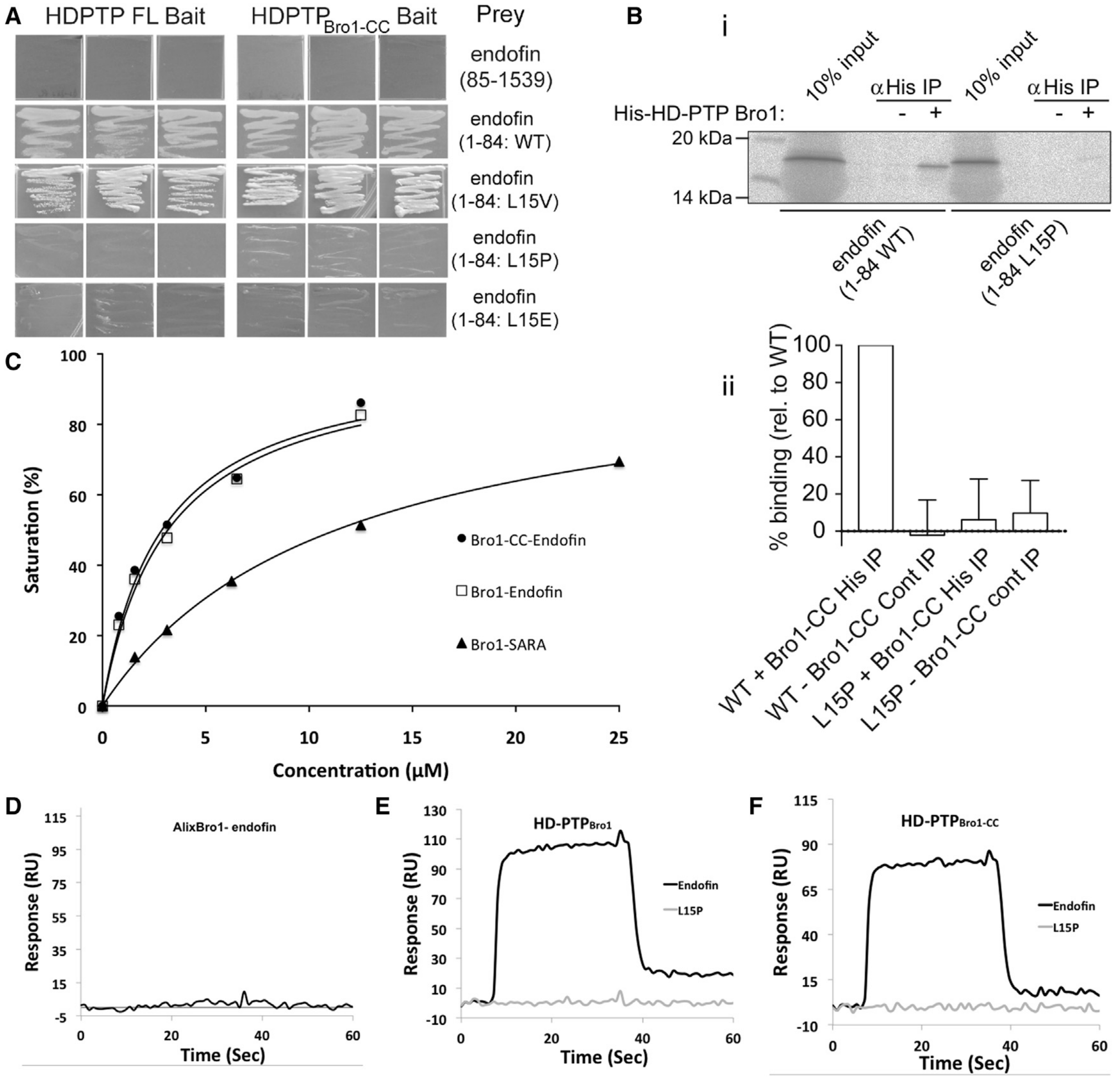

Figure 2. Mapping the HD-PTP Binding Interactions with SARA and Endofin

(A) Y2H assays between endofin(1–84) constructs or endofin(85–1,539) and HD-PTP$_{FL}$ or HD-PTP$_{Bro1-CC}$ shows that binding is restricted to the endofin N terminus and that endofin L15 is important for binding. Data are from a representative experiment performed in triplicate.

(B) In vitro translated endofin$_{1-84}$-strep, but not endofin(1–84/L15P)-strep, is co-immunoprecipitated with bacterially expressed His$_6$-HD-PTP$_{Bro1-CC}$ after an incubation with anti-His$_6$ antibody; (i) is an example experiment, (ii) is quantitation from three independent experiments (mean ± SD).

(C) Biosensor binding isotherms for the HD-PTP$_{CC}$ and HD-PTP$_{Bro1-CC}$ binding to endofin and SARA 22-mer peptides, used to determine the binding affinities (HD-PTP$_{Bro1}$ and HD-PTP$_{Bro1-CC}$ binding to the endofin peptide have K_D 3.1 ± 0.1 μM and 2.7 ± 0.1 μM, respectively, and HD-PTP$_{Bro1}$ has K_D 11.3 ± 0.3 μM to SARA).

(D) Biosensor sensogram showing the lack of binding of the endofin peptide to Alix$_{Bro1}$.

(E and F) Sensograms showing that mutation of endofin L15 to proline abolishes binding to HD-PTP$_{Bro1}$ (E) and HD-PTP$_{Bro1-CC}$ (F) compared with the control experiment using the wild-type endofin peptide.

L202D mutant, as judged by the SPR signal versus the buffer control (Figure 3B). These data suggest that endofin binds to a site on HD-PTP that overlaps with its CHMP4 binding site. We therefore examined whether endofin competes with CHMP4B for binding. Interaction of CHMP4B with HD-PTP$_{Bro1-CC}$ was essentially abolished by addition of the endofin peptide, but not by an endofin peptide containing the substitution L15P (Figure 3C). These data demonstrate that endofin and CHMP4B share binding to the conserved hydrophobic site in the HD-PTP Bro1 domain (referred to hereafter as the "common site").

For Alix and Brox, it has been shown that the C-terminal helix of CHMP4B is responsible for binding to the common site in the Bro1 domain (McCullough et al., 2008; Mu et al., 2012), but this has not been formally demonstrated for HD-PTP. Y2H experiments confirmed that this region is also relevant for binding to HD-PTP, since CHMP4B lacking the C-terminal six amino acids (C4BΔCT) did not bind Alix$_{Bro1-V}$ or HD-PTP$_{Bro1-CC}$, while full-length CHMP4B did (Figure 3D). CoIP confirmed that CHMP4B-FL, but not C4BΔCT, binds to HD-PTP$_{Bro1-CC}$ (Figure 3E). Next, peptides designed from the C-terminal 18–20

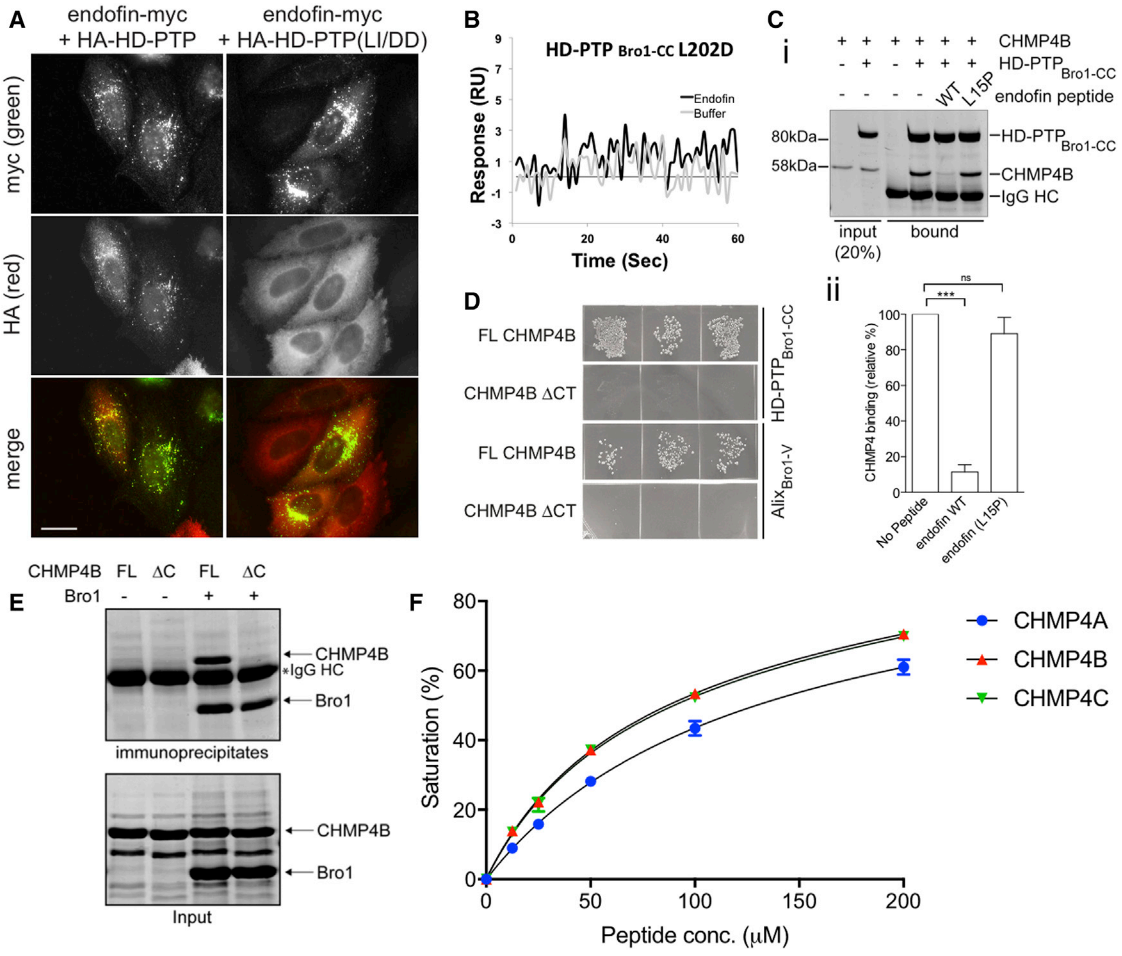

Figure 3. Endofin and CHMP4B Compete for Binding to the Bro1 Domain of HD-PTP

(A) In contrast to wild-type HA-HD-PTP, HA-HD-PTP(I202D, L206D) is not recruited to endosomes enriched with endofin-myc when co-transfected. Scale bar, 10 μm.

(B) Binding of the endofin peptide is impaired by the I202D mutation, as shown in the biosensor sensogram compared with the buffer control.

(C) Bacterially expressed GST-CHMP4B is co-immunoprecipitated with bacterially expressed His$_6$-HD-PTP$_{Bro1\text{-}CC}$ after an incubation with anti-His$_6$ antibody. This is prevented by inclusion of endofin$_{1\text{-}22}$ (WT), but not by endofin$_{1\text{–}22(L15P)}$. (i) is an example experiment, (ii) is quantitation from three independent experiments (mean ± SD). Statistical analysis was performed using the unpaired t test with Welch's correction: ***$p < 0.001$; ns, not significant.

(D) Y2H assays using CHMP4 baits and HD-PTP or Alix prey constructs show that interaction between CHMP4B and both HD-PTP$_{Bro1\text{-}CC}$ and Alix$_{Bro1\text{-}V}$ requires the CHMP4B C-terminal six amino acids.

(E) Co-immunoprecipitation of bacterially expressed proteins with anti-His antibody shows that binding to His$_6$-HD-PTP$_{Bro1\text{-}CC}$ is prevented by deletion of the C-terminal six amino acids of GST-CHMP4B. Representative of three independent experiments.

(F) Biosensor binding isotherms for the HD-PTP$_{Bro1}$ to the CHMP4 peptides shows similar affinity with K_D of 132 ± 7.0 μM, 85 ± 2 μM, and 88 ± 1 μM for CHMP4A, B, and C, respectively. Biosensor experiments were performed at least three times. Error bars denote SEM.

residues of CHMP4A, CHMP4B, and CHMP4C were evaluated for binding to HD-PTP$_{Bro1}$ (Figure 3F). Interestingly, CHMP4 peptides showed much lower affinity to HD-PTP$_{Bro1}$ compared with the SARA/endofin peptides, with K_D values of 132 ± 7 μM for CHMP4A, 85 ± 2 μM for CHMP4B, and 89 ± 1 μM for CHMP4C. Altogether, our binding studies suggest that SARA/endofin could compete effectively with CHMP4 for binding to HD-PTP, based on their higher observed affinity and coIP experiments.

Crystal Structures of HD-PTP$_{Bro1}$-CHMP4A, B, C and HD-PTP$_{Bro1}$-SARA/Endofin Complexes

The biochemical and binding studies suggested that unknown key molecular determinants may be responsible for the higher binding affinity of SARA/endofin peptides over CHMP4 peptides, despite the apparent overlap of their binding sites. The binding selectivity exhibited by SARA/endofin toward HD-PTP compared with Alix is also surprising, given the high conservation at the CHMP4 binding site (Kim et al., 2005) (Doyotte et al., 2008); (Mu et al., 2012) (McCullough et al., 2008). To resolve this paradox, we decided to investigate each of the specific interactions by X-ray crystallography. We determined high-resolution crystal structures of HD-PTP$_{Bro1}$ alone (*apo*-HD-PTP$_{Bro1}$), HD-PTP$_{Bro1}$ in complex with the C-terminal peptides of CHMP4A, B, and C, respectively, and HD-PTP$_{Bro1}$ in complex with the N-terminal peptides of SARA and endofin (Figure 4 and Table 1). The *apo*-HD-PTP$_{Bro1}$ structure matches with previously reported

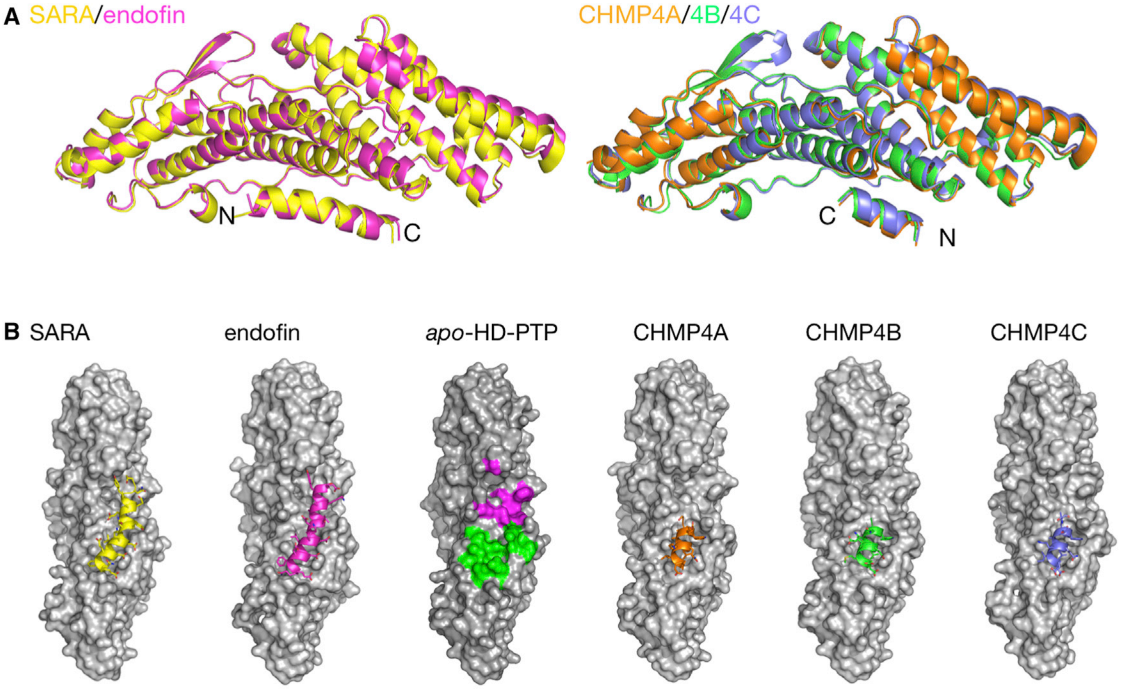

C

SARA/endofin binding

CHMP4 binding

HD-PTP	E137	K141	L189	K192	R198	L202	L206	A336	L338	V339	F62	H125	V142	T145	H146	L342	D348
Alix	D143	K147	F199	K202	M208	I212	L216	A335	L337	V338	E62	A141	I148	K151	H152	T341	I347
Brox	E137	K141	T195	R198	H204	L208	L212	N347	L350	V351	E55	Y122	E142	R145	S146	L354	P360

Common site — Specific S-site

Figure 4. Crystallographic Structures of HD-PTP$_{Bro1}$ with Endosomal Effectors

(A) Ribbon diagrams showing the structures of the complexes of HD-PTP$_{Bro1}$ with SARA (yellow) and endofin (fuschia) peptides on the left, and with the three CHMP4 isoform peptides (4A, orange; 4B, green; 4C, purple) on the right. All peptides form helical structures and bind to the concave region of the Bro1 domain.

(B) Surface representations of the six structures determined in this study, showing the peptides as α-helical ribbons and the Bro1 domains as molecular surfaces (gray). The colored regions in the *apo*-HD-PTP structure highlight the common-CHMP4 binding site (green) and the specific S site (magenta), only utilized by the SARA/endofin peptides. Peptides are color coded as in (A).

(C) Residues in the Bro1 domain involved in binding to the effector peptides at the common and specific sites in the structures shown in (B). Conserved residues between Bro1 proteins are shown in black and substitutions are in red. The different binding sites are labeled (common site and S site) and the extent of the interface between CHMP4 peptides (green) and SARA/endofin (fuschia) is highlighted.

structures of HD-PTP$_{Bro1}$ (PDB: 3RAU and 5CRU), with a root-mean-square deviation (RMSD) of 0.70–0.76 Å.

All five peptides form amphipathic α helices that bind to the concave hydrophobic surface of the Bro1 domain, confirming the overlap suggested by the biochemical studies (Figures 4A and 4B). However, the binding interface for SARA/endofin peptides is clearly larger than that for the CHMP4 peptides, extending beyond the common site toward a specific pocket (S site), which is poorly conserved in Alix or Brox (Figures 4B and 4C). For the CHMP4 peptides only 11 residues are clearly visible in the electron density maps, whereas 20 residues are visible for the SARA and endofin peptides (Figure S2).

All three CHMP4 peptides bind in the same way across helices H5, H6, and H7 of HD-PTP$_{Bro1}$ (Figure 5A), and form similar van der Waals contacts with a number of conserved residues, including the critical L202/I206 pair (Table 2 and Figure 5B). Additional contacts occur with hydrophobic residues at the proline-rich C-terminal region of HD-PTP$_{Bro1}$ (Figure 5C; see Table 2 for a list of all interactions). The main CHMP4 anchoring residues are a Leu and a Trp present in all three isoforms (L217 and W220 in CHMP4A and CHMP4B, L228/W231 in CHMP4C), which contact HD-PTP$_{Bro1}$ residues L189, L202, and I206 from two hydrophobic pockets between H5 and H6 and H6 and H7 (Figure 5B). Electrostatic/hydrogen bond interactions also contribute to the binding interface, between conserved acidic and polar residues on the CHMP4 peptides and R198 on HD-PTP$_{Bro1}$ (Table 2 and Figure 5D), and the indole nitrogen of W220/W231 and E137 on HD-PTP$_{Bro1}$ (Figure 5B).

The mode of binding of CHMP4 peptides to HD-PTP$_{Bro1}$ is similar to that reported for the complexes of CHMP4 peptides

Table 1. Data Collection and Structure Refinement Statistics

	SARA	Endofin	Apo-HD-PTP	CHMP4A	CHMP4B	CHMP4C
Data Collection						
Space group	P 1	P 1	P 2_1	P 1	P 2_1	P 1
Cell dimensions						
a, *b*, *c* (Å)	66.3, 70.4, 83.5	43.8 64.7 70.6	69.5 65.0 81.5	71.5 73.7 79.4	68.4 64.9 81.2	66.4 70.8 79.3
α, β, γ (°)	111.5 90.1 93.0	101.4 97.2 102.2	90.0 91.3 90.0	114.4 90.3 104.2	90.0 90.2 90.0	90.5 98.2 106.8
Molecules. per a.u. (peptide)	4 (2)	2 (2)	2	4 (4)	2 (1)	4 (4)
Resolution (Å)	62.58–2.25	41.19–1.77	28.49–1.87	28.41–2.5	50.68–1.7	49.92–2.0
R_{merge}	0.10 (0.33)	0.05 (0.29)	0.12 (0.88)	0.13 (0.80)	0.04 (0.25)	0.06 (0.23)
$I/\sigma I$	6.8 (2.8)	10.2 (3.2)	9.2 (2.0)	7.9 (1.9)	18.6 (4.1)	12.0 (4.2)
Completeness (%)	95.9 (93.8)	96.5 (93.6)	99.3 (97.3)	93.1 (84.8)	98.7 (95.2)	95.9 (94.2)
Redundancy	2.1 (2.2)	2.2 (2.2)	4.1 (4.0)	3.5 (3.5)	3.3 (2.6)	2.2 (2.1)
Refinement						
Resolution (Å)	2.25	1.77	1.87	2.50	1.70	2.00
No. of reflections	63,813 (6,239)	69,285 (6,748)	60,088 (5,842)	45,700 (4,178)	77,352 (7,420)	88,589 (8,712)
R_{work}/R_{free}	0.20/0.23	0.16/0.19	0.17/0.22	0.18/0.23	0.17/0.20	0.18/0.20
No. of atoms						
Protein	11,377	5,747	5,704	11,667	5,742	11,464
Peptide	348	270	NA	356	90	330
Water	584	531	542	203	676	781
B factors						
Protein	35.6	30.8	27.1	48.4	23.8	37.8
Peptide	41.4	28	NA	79.9	28.9	44.8
Water	41.7	39.9	34.9	40.3	32.8	41.9
RMSDs						
Bond lengths (Å)	0.006	0.005	0.013	0.003	0.003	0.002
Bond angles (°)	1.05	0.64	1.26	0.66	0.58	0.53

Each structure was determined from one crystal. Values in parentheses represent highest-resolution shell.

with Alix or Brox Bro1 domains (McCullough et al., 2008; Mu et al., 2012). However, some significant differences are observed. One difference is the role of R198 in HD-PTP$_{Bro1}$. In Alix this position is occupied by M208, which forms van der Waals contacts with CHMP4 L217 but does not form specific interactions with acidic residues in the CHMP4 peptides (McCullough et al., 2008). Another important difference is the availability of the two hydrophobic pockets at the CHMP4 binding site, which are fully exposed in the Alix and Brox Bro1 domains (McCullough et al., 2008; Mu et al., 2012). In the *apo*-HD-PTP$_{Bro1}$ structure these are blocked by the side chains of K141, K192, and R198 (Figure 5E). Thus, binding of CHMP4A/B/C to HD-PTP$_{Bro1}$ requires rearrangement of these side chains to accommodate the critical CHMP4 Leu and Trp residues (Figure 5E).

Structural Basis for Selective Interaction of HD-PTP with SARA and Endofin

As predicted (Figure 1A), the 22-mer SARA/endofin peptides form amphipathic α helices in their complexes with HD-PTP$_{Bro1}$. They occupy a larger binding site than the CHMP4 peptides, partially overlapping with the common site across H5, H6, and H7, but extending further into the S site between H3 and H5 (Figures 4 and 6A). SARA/endofin peptides bind to HD-PTP$_{Bro1}$ in the opposite orientation to the CHMP4 peptides, with their C termini sitting above helix H7 and their N termini in the S site (Figure 6A).

The SARA/endofin-HD-PTP$_{Bro1}$ binding interface is largely hydrophobic and involves residues F5, A9/V9, L12, L16, and F19 present in both peptides (Figure 1A and Table 2). SARA/endofin peptides lack the Trp residue conserved in the CHMP4 peptides, but L12 occupies the analogous position, binding to one of the hydrophobic pockets and interacting with L189 in HD-PTP$_{Bro1}$ (Figure 6B). The smaller size of Leu versus Trp means that fewer rearrangements of this pocket are necessary to accommodate the SARA/endofin versus CHMP4 peptides. Rearrangement mostly consists of a displacement of the R198 side chain and a slight movement of the K141 and K192 side chains (Figure 5E). Leu16 accommodates itself into the second hydrophobic pocket and interacts with L202/I206 in the endofin-HD-PTP$_{Bro1}$ complex and I206 in the SARA-HD-PTP$_{Bro1}$ complex (Figure 6D), consistent with the importance of these residues for SARA/endofin binding to HD-PTP.

The crystal structures explain the impaired binding to HD-PTP$_{Bro1}$ shown by L15P and L15E mutants of endofin (Figures 2 and 3). L15 forms hydrophobic interactions with L338 at the proline-rich C-terminal region of HD-PTP$_{Bro1}$ (Table 2). Substitution by Glu would disrupt these interactions and also clash with L338, whereas substitution by Pro may cause a disruption of the

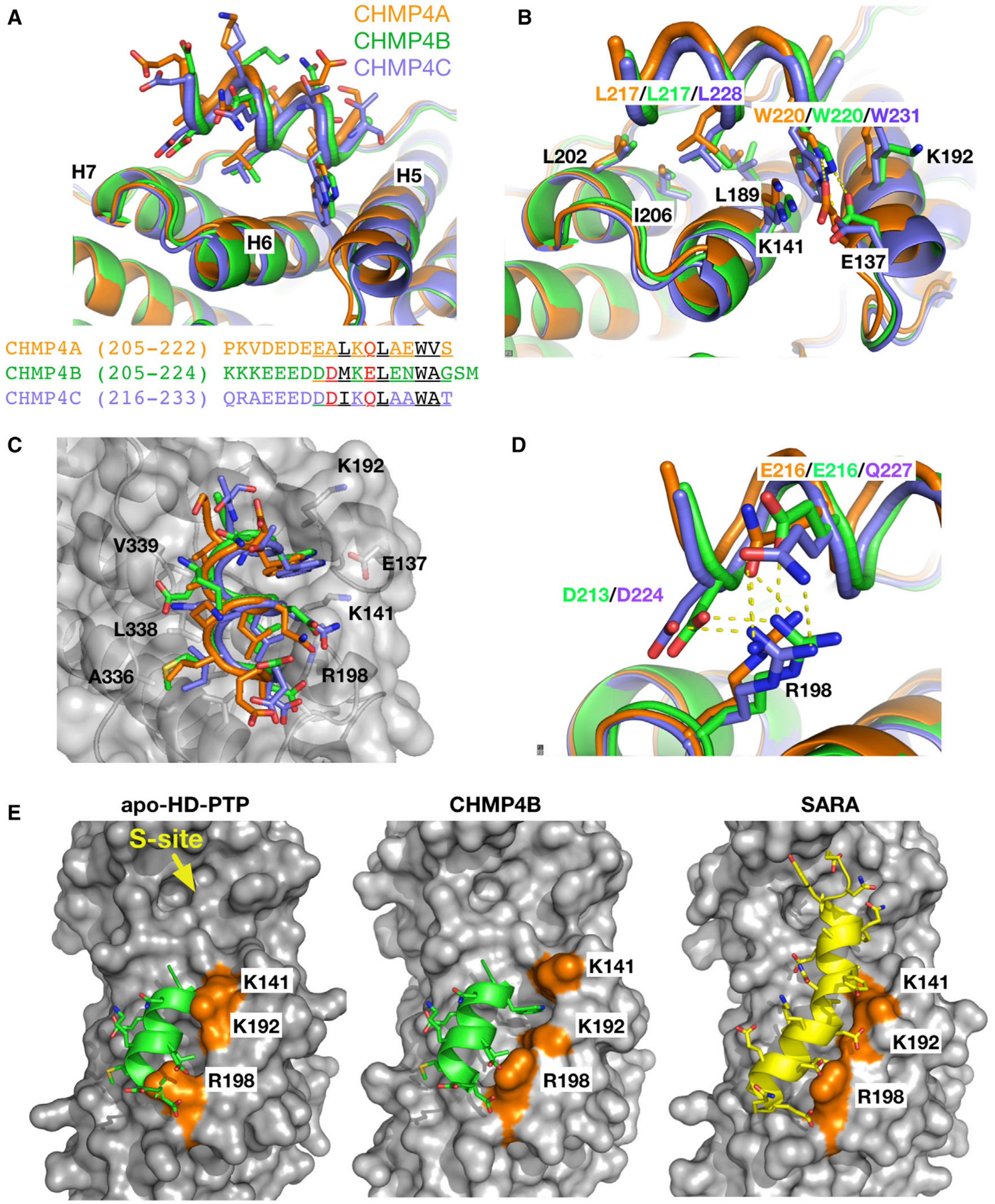

Figure 5. Analysis of the HD-PTP$_{Bro1}$-CHMP4 Interfaces

(A) Ribbon representation of the structures of the three HD-PTP$_{Bro1}$-CHMP4 complexes (4A, orange; 4B, green; 4C purple) showing the interface at the common binding site on the hydrophobic concave region of HD-PTP$_{Bro1}$. Peptide side chains are shown as sticks. The sequences of each peptide used in the crystallization are shown below, where residues visible in the structures are underlined, hydrophobic residues are in black, and acidic/polar residues are in red.

(B) Detail of the binding site showing residues in HD-PTP$_{Bro1}$ (black labels) that form interactions with the conserved Leu and Trp residues in the peptides (labeled according to color code for each peptide).

(C) Surface representation of HD-PTP$_{Bro1}$ with the three peptides superimposed showing the binding into the two hydrophobic pockets and the HD-PTP$_{Bro1}$ residues involved both at the C-terminal region (left side) and at the common site (right side) labeled in black.

(D) Detail of the electrostatic interactions between HD-PTP$_{Bro1}$ R198 and the acidic and polar residues in the CHMP4 peptides.

(E) Surface representation of the structures of *apo*-HD-PTP$_{Bro1}$ with the CHMP4B peptide superimposed (left), HD-PTP$_{Bro1}$-CHMP4B complex (center), and HD-PTP$_{Bro1}$-SARA complex (right). Colored in orange and labeled in black are the residues that block the hydrophobic pockets in the *apo* structure and that undergo conformational rearrangements in the complexes with the peptides. In contrast, the S-site pocket is readily available in the *apo* structure.

Table 2. Residues in Direct Interaction—van der Waals Contacts, Hydrogen Bonds—in the Crystal Structures of HD-PTP$_{Bro1}$ Complexes with Different Peptides from CHMP4, SARA, or Endofin

HD-PTP$_{Bro1}$	CHMP4A	CHMP4B	CHMP4C	SARA	Endofin
F62	–	–	–	F5	F5
H125	–	–	–	F5	F5
V142	–	–	–	F5<br>A9	F5<br>V9
T145	–	–	–	F5<br>A9	F5<br>V9
H146	–	–	–	F5	F5
L342	–	–	–		A8
D348	–	–	–	Y4	Y4
E137	W220 Ne	W220Ne	W231Ne	D13	D13
K141	W220	W220	W231	D13	D13
Q148	V221	A221	A232	–	–
L189	L217 W220 V221	L217 W220 A221	L228 W231 A232	L12 L16	L12 L16
K192	W220	W220	W231	D13	D13
R198	Q216	D213 E216	D224 Q227	D17 E20	D17 E20
L202	L214 L217	M214 L217	I225 L228	F19	F19
R205	–	–	–	F19	F19
I206	L217	L217	L228	L16	L16
A336	L214	M214	–	F19	F19
L338	L214 L217	M214 L217	I225 L228	V15 F19	L15 F19
V339	V221	A221	A232	–	–

helical structure and potentially result in the loss of several interactions at either side.

Phenylalanine residues (F5 and F19), at both ends of the α helices, anchor SARA/endofin peptides into the binding site (Figure 6C). Phe19 forms multiple interactions at the common site, including with L202/I206 (Figure 6B), while F5 fits into the S site forming several van der Waals contacts (Table 2 and Figure 6D). SARA/endofin peptides also contain a cluster of acidic residues that form hydrogen bonds with HD-PTP (E137, K141, K192, and R198), thus contributing significantly to the binding affinity (Table 2 and Figure 6E).

The most important difference between the mode of binding of CHMP4 and SARA/endofin peptides to HD-PTP are the new interactions at the S site, which are key to providing specificity and selectivity (Figure 6D). Significantly, the S site appears fully open and accessible in the structure of the *apo*-HD-PTP$_{Bro1}$, and no major conformational changes are observed upon binding of SARA/endofin peptides (Figure 5E). Most HD-PTP$_{Bro1}$ residues involved in interactions at the S site, notably F62, H125, and T145, are not conserved in Alix or Brox (Figure 4). In particular, the position of T145 is occupied by K151 in Alix and R145 in Brox, with their side chains imposing steric hindrance that blocks binding beyond this point (Figure 7A). In HD-PTP$_{Bro1}$, the smaller side chain of T145 allows the additional two helical turns at the N terminus of the SARA/endofin peptides to reach into the S site. Therefore, T145 acts as a gatekeeper between the common site and the new S site, as well as participating in binding (Table 2 and Figure 6D). T145 has also been reported to be important for STAM2 binding to HD-PTP (Lee et al., 2016).

Another key interaction in the S site is the hydrogen bond between the hydroxyl group of Y4 in SARA/endofin and D348 in HD-PTP$_{Bro1}$ (Figure 6D). This aspartic acid is not present in Alix or Brox, where an Ile or Pro residue is found instead (Figure 4). Tyrosine 4 is contained in the N-terminal motif, MXXYF, conserved in both SARA and endofin (Figure 1A), but absent from CHMP4. Indeed, a shorter endofin peptide lacking 1-MDSYF-5 showed poor binding (>1.5 mM) to HD-PTP$_{Bro1}$ or HD-PTP$_{Bro1\text{-}CC}$ (Figure 7B), confirming the importance of the Y4 and F5 interactions.

Overall, the SARA/endofin peptides form an extensive binding interface that encompasses both the CHMP4 site and the S site, resulting in accessible buried surface in HD-PTP$_{Bro1}$ ranging from 762 to 780 Å^2, compared with 468 to 473 Å^2 for the CHMP4 peptides. This larger interface, together with the additional new interactions at the S site, explains the higher binding affinity that we observe (Figure 2). The structures of HD-PTP$_{Bro1}$ with SARA/endofin peptides therefore rationalize their higher affinities for HD-PTP over CHMP4, their ability to compete with CHMP4 for binding to the Bro1 domain, and their selectivity for HD-PTP versus Alix.

Mutational Analysis of the HD-PTP$_{Bro1}$-SARA/Endofin Binding Interface

Based on the structural analysis we anticipated that F62, H125, and T145 on HD-PTP$_{Bro1}$ would be important for the specific binding to SARA/endofin (Figure 7C). To test their importance, we mutated these residues to their cognate residues in Alix (F62E, H125A, and T145K). Two further mutations, R69L and R198M, were also assessed. R198 contributes to electrostatic interactions at the conserved CHMP4 site. R69 lies within the

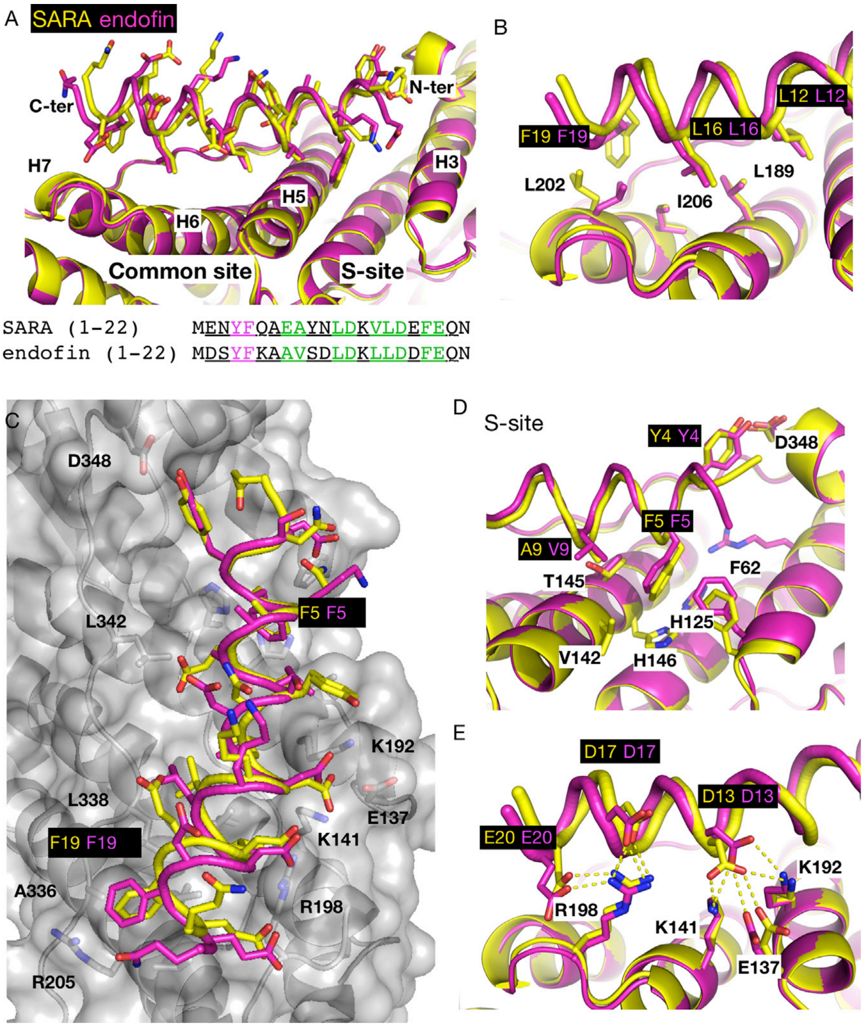

Figure 6. Analysis of the Interface of HD-PTP$_{Bro1}$ with SARA and Endofin

(A) Ribbon representation of the structures of the HD-PTP$_{Bro1}$-SARA (yellow) and HD-PTP$_{Bro1}$-endofin (fuschia) complexes. The binding interface is extensive and covers the common CHMP4 binding site and a unique specific S site. Helices in the binding site are labeled. Below are the sequences of both peptides used in the crystallization where residues visible in the structures are underlined, residues that form interactions at the common site are in green, and those that bind to the S site are in magenta.

(B) Detail of the HD-PTP$_{Bro1}$-SARA/endofin interface at the common site, showing the interaction of the conserved L12, L16, and F19 residues from SARA/endofin (color labels on black background) with the critical L202/I206 (Doyotte et al., 2008) and L189 residues from HD-PTP$_{Bro1}$ (black labels).

(C) Surface representation of HD-PTP$_{Bro1}$-SARA structure with the SARA/endofin peptides superimposed. Labeled in black are the HD-PTP$_{Bro1}$ residues involved in interactions with the peptides at the C-terminal region (left side) and the common site (right side) of the Bro1 domain, labeled in black. The two conserved Phe residues in SARA/endofin are labeled according to the color code of each peptide.

(D) Detail into the S site showing all residues involved in interactions. Color scheme as in (B).

(E) Detail of the electrostatic interactions between basic residues in HD-PTP and the acidic residues in SARA/endofin. Color scheme as in (B).

S site and although it does not show any obvious interactions, its proximity to the acidic E2/D2 in SARA/endofin might contribute to binding. We then assessed endofin binding to all these mutants in cell-based assays, alongside L202D as a control. Lysates from cells expressing HA-HD-PTP$_{Bro1-CC}$ (wild-type and mutants) were immunoprecipitated with anti-HA beads and immunoblotted for endogenous full-length endofin (Figure 7D). Binding was also tested in vitro by co-translating endofin(1–84) and His$_6$HD-PTP$_{Bro1-CC}$, then immunoprecipitating with anti-His (Figure S3). In both instances, binding to endofin was virtually abolished by T145K and L202D mutations, and was also substantially reduced by H125A and F62E. The R69L and R198M mutations also slightly reduced binding to endofin (Figures 7D and S3). Binding of full-length CHMP4B to HD-PTP$_{Bro1-CC}$ was unaffected by the T145K mutation (Figure 7E), confirming that access to the S site is not required for CHMP4 binding but is critical for interaction with endofin and presumably SARA as well.

In summary, our structural analysis of the five HD-PTP$_{Bro1}$ complexes with peptides provides a molecular basis to explain the high affinity of SARA/endofin for HD-PTP$_{Bro1}$ compared with CHMP4, as well as the selectivity of SARA/endofin for HD-PTP over other Bro1 proteins, such as Alix. Furthermore we show that, unlike the S site, the CHMP4 binding site is not readily available in the *apo*-HD-PTP structure, requiring conformational rearrangements. Altogether, these findings support a competition-based mechanism for controlling access of ESCRT-III to the HD-PTP Bro1 domain.

DISCUSSION

The decision of whether activated receptors are recycled from the early endosome, retained, or degraded via the MVB pathway is vital for determining cellular signaling responses. Furthermore, this switch is decisive in integrating signaling between multiple receptor systems that converge at the endosome, such as mitogenic signaling, integrins, and TGFβ/BMP signaling (von Zastrow and Sorkin, 2007). A central question therefore is how the membrane-remodeling, pro-degradative function of ESCRT-III is controlled at the early endosome in response to different stimuli to determine receptor fate. In this study we identify novel interactions of SARA and endofin, endocytic regulators of TGFβ/BMP signaling, to HD-PTP, and show that they compete with the ESCRT-III subunit CHMP4 for binding to the HD-PTP Bro1 domain.

The ability of these endosomal proteins to influence HD-PTP engagement with ESCRT-III identifies HD-PTP as a key

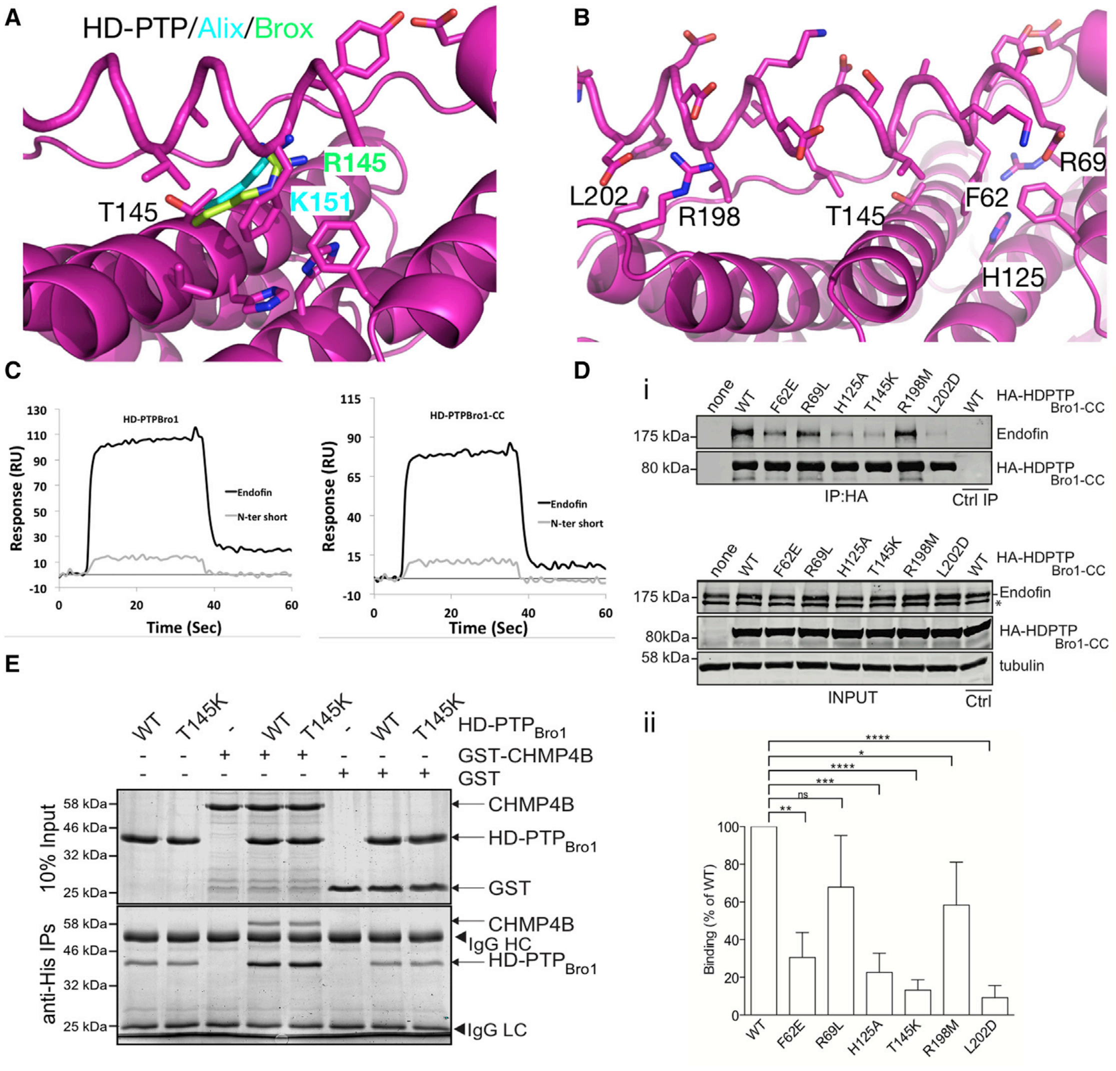

Figure 7. Mapping the Interface at the S Site

(A) Detail at the S site of HD-PTP$_{Bro1}$-endofin complex showing the position of T145, a key residue at the binding interface. The cognate residues on the related Bro1 proteins (K151 in Alix in cyan; R145 in Brox in green) are larger and clearly would clash with the endofin peptide, explaining the binding selectivity observed.

(B) Detail of the HD-PTP$_{Bro1}$-endofin binding interface showing the position of the residues used in the mutagenesis evaluation, labeled black.

(C) Biosensor binding studies confirmed the importance of the conserved MXXYF region at the N terminus of the SARA and endofin peptides. An endofin peptide lacking these residues shows poor binding to HD-PTP$_{Bro1}$ and HD-PTP$_{Bro1\text{-}CC}$ as shown in contrast to the wild-type endofin peptide.

(D) HA-HD-PTP$_{Bro1\text{-}CC}$ or the indicated mutants were expressed in cells and anti-HA immunoprecipitates were probed with anti-endofin antibody. Left panel shows an example experiment, representative of three independent experiments. Inputs are shown in the bottom panel and anti-HA Immunoprecipitations (IP) are shown above. The asterisk denotes a likely endofin cleavage product lacking the N terminus, or cross-reacting species recognized by the endofin antibody. The species is present in cell lysates but not in anti-HA IPs. Right panel shows quantitation from four independent experiments (mean ± SD). Statistical analysis was performed using the unpaired t test with Welch's correction: ****$p < 0.0001$, ***$p < 0.001$, **$p < 0.01$, *$p < 0.05$; ns, not significant.

(E) Co-immunoprecipitation of bacterially expressed GST, or GST-CHMP4B combined with His-HD-PTP, shows that binding to GST-CHMP4B is not affected by mutation of T145 to K in His$_6$-HD-PTP$_{Bro1\text{-}CC}$. Representative of three independent experiments.

modulator of the MVB sorting switch, consistent with its tumor-suppressor function (Manteghi et al., 2016). Understanding at the molecular level how HD-PTP interacts with each endosomal effector is critical for elucidating how the switch works. To this end, we have determined high-resolution crystal structures of HD-PTP$_{Bro1}$ in complex with relevant binding peptides from SARA and endofin as well as each of the three CHMP4 isoforms A, B, and C. Crystallographic analysis combined with mutagenesis and binding studies provide a molecular basis for the high affinity and selectivity of SARA/endofin for HD-PTP.

The structures of HD-PTP$_{Bro1}$ in complex with the respective peptides from these five effectors reveal a common binding site at the concave hydrophobic surface of the Bro1 domain. This common site is conserved in other Bro1 proteins and contains the critical L202 and I206 residues, previously identified as essential for HD-PTP function (Ali et al., 2013; Doyotte et al.,

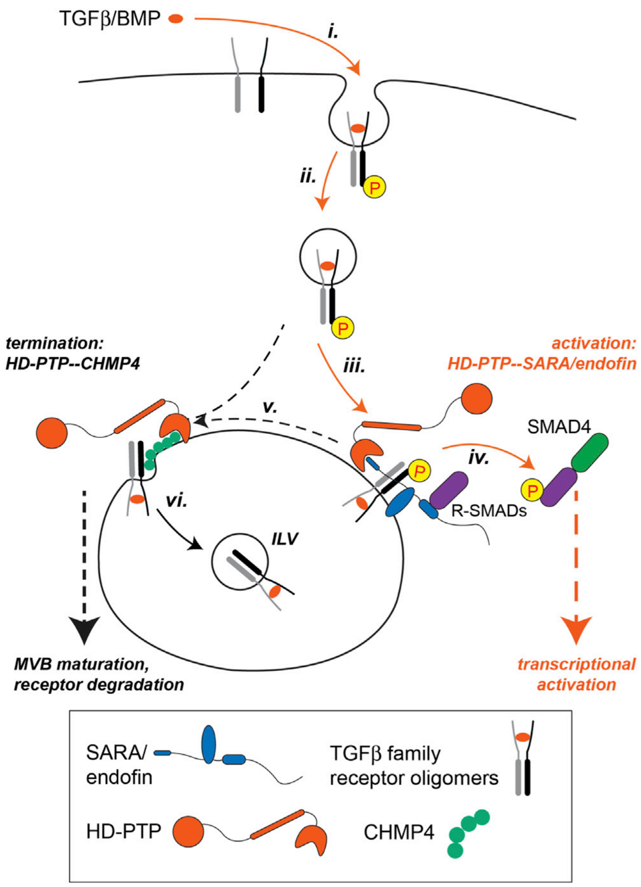

Figure 8. Model of Endocytic Regulation of TGFβ/BMP Signaling

Activation of TGFβ family receptors by ligand binding (i) leads to formation of receptor oligomers, receptor phosphorylation, and internalization (ii). As the activated receptor complexes are targeted to the endosome they recruit SARA/endofin (iii), which bind the HD-PTP Bro1 domain to prevent receptors engaging the ESCRT pathway. SARA/endofin recruit R-Smads to the receptor complex (iv), leading to R-Smad phosphorylation and subsequent binding to the co-Smad, Smad4, allowing transcriptional regulation. Dephosphorylated receptors either fail to engage with or are released from SARA/endofin, allowing HD-PTP to then recruit CHMP4/ESCRT-III (v) to target receptors to ILVs (vi) and ultimately toward lysosomal degradation. Interaction between HD-PTP and ESCRT-0 may provide an intermediate step prior to CHMP4 recruitment, and it is likely that receptor ubiquitination promotes its engagement with ESCRTs. These details are not shown.

2008; McCullough et al., 2008; Mu et al., 2012). However, the two hydrophobic pockets, critical for binding of the CHMP4 peptides, are occluded in the *apo*-HD-PTP$_{Bro1}$ structure, requiring several side-chain conformational changes to accommodate the peptides. This may contribute to maintaining HD-PTP in an inactive status by minimizing interactions between cytosolic pools of CHMP4 and HD-PTP. In contrast, these pockets are fully open in the *apo* structures of Alix and Brox (McCullough et al., 2008; Mu et al., 2012).

Similar conformational rearrangements are observed in the complex of HD-PTP with a STAM2 peptide (Lee et al., 2016), which also shows higher binding affinity than CHMP4. Is it therefore feasible that binding of STAM2 may serve as a primer for the subsequent binding of HD-PTP to ESCRT-III/CHMP4, by opening the hydrophobic site and thus facilitating exchange with the lower-affinity binder, CHMP4. Such a scenario would contribute to the sequential interaction of HD-PTP with ESCRTs, as cargo is first sorted by ESCRT-0 and ultimately incorporated into the developing ILV by ESCRT-III (Ali et al., 2013).

A major finding from the structural analysis is that the endofin and SARA binding site expands from the common site to a neighboring specific pocket (S site), not occupied by the CHMP4 peptides and fully accessible in the *apo*-HD-PTP$_{Bro1}$ structure. The extended interface and the availability of the S site explain why these peptides bind to HD-PTP with at least 30-fold higher affinity than CHMP4 peptides, and effectively compete with CHMP4 for binding. Key HD-PTP residues (F62, H125, and T145) involved in SARA/endofin interactions at the S site are not conserved in Alix or Brox, thus explaining the observed selectivity. Interestingly, T145, which sits at the edge of the S site, is also important for STAM2 binding to HD-PTP (Lee et al., 2016) but is irrelevant for CHMP4B interaction (this study and Lee et al., 2016). In contrast, L202, which is located at the center of the hydrophobic binding site, is essential for binding to CHMP4 as well as to SARA/endofin.

These results suggest that specific interactions at the S site confer higher affinity, providing a competitive binding advantage over ESCRT-III. This, together with the structural rearrangements required for CHMP4 to occupy the common site, would effectively prevent endosome-associated HD-PTP engaging CHMP4 in a constitutive manner, i.e., before cargo is committed to ILV entry down the degradative route. Hence, under appropriate environmental conditions, TGFβ/BMP signaling may take precedent, with SARA/endofin hijacking HD-PTP at the endosome and blocking the MVB pathway to delay degradation of TGFβ/BMP receptors. When the environment favors TGFβ/BMP receptor degradation, STAM2 may be suited to occupy an intermediate step, helping the disengagement of SARA/endofin and leading to the recruitment of CHMP4 polymers to HD-PTP (Ali et al., 2013). The precise order of these binding reactions, and the environmental conditions at the endosome under which each binding partner is favored, await detailed further investigation.

We propose a model for how HD-PTP might control TGFβ/BMP signaling by integrating it with receptor trafficking (Figure 8). The specific binding mode of endofin and SARA to HD-PTP to prevent recruitment of CHMP4 is central to this mechanism. Specifically, SARA/endofin binding to HD-PTP may delay ESCRT recruitment and the subsequent MVB sorting and degradation of internalized TGFβ/BMP receptors. This would allow longer residency of the activated receptors at the endosome in order to phosphorylate Smad proteins and trigger downstream signaling to the nucleus. Of particular significance, by binding to HD-PTP via their N termini, endofin and SARA will remain fully functional as Smad signaling scaffolds. Presumably, release of activated Smads may then induce disengagement of SARA/endofin from HD-PTP, and allow its subsequent recruitment of ESCRTs and incorporation of TGFβ receptors into the MVB pathway for degradation. Future experiments will test this hypothesis.

In summary, understanding at the molecular level the interactions of this important endosomal regulator, HD-PTP, with its effectors opens new avenues to investigate its role in controlling endocytic signaling pathways.

STAR★METHODS

Detailed methods are provided in the online version of this paper and include the following:

- KEY RESOURCES TABLE
- CONTACT FOR REAGENT AND RESOURCE SHARING
- METHOD DETAILS
  - Expression Constructs for Heterologous Expression
  - Protein Purification
  - Biosensor Binding Studies
  - Crystallization
  - Data Collection and Structure Determination
  - Antibodies
  - Yeast Two Hybrid Analysis
  - Cell Culture, Transfections and Microscopy
  - Immunoprecipitations from Cell Lysates
  - In Vitro Translation and Binding Assays
- QUANTIFICATION AND STATISTICAL ANALYSIS
  - Biosensor Binding Studies
  - Immunoprecipitations and Binding Assays
- DATA AND SOFTWARE AVAILABILITY
  - Accession Numbers

SUPPLEMENTAL INFORMATION

Supplemental Information includes three figures and one table and can be found with this article online at http://dx.doi.org/10.1016/j.str.2017.05.005.

AUTHOR CONTRIBUTIONS

D.G. prepared protein samples and crystallized the proteins. C.L. collected X-ray diffraction data, and solved and refined the crystal structures. A.P.M. and D.G. collected SPR data and A.P.M. analyzed data. L. Walker performed the yeast two-hybrid and coIP experiments. S.T. performed yeast two-hybrid experiments. L. Wunderley performed mutagenesis and coIP experiments. P.W. performed, supervised, and analyzed cell biology and biochemistry data. L.T. designed and supervised the project, and analyzed the structures. L.T and P.W. wrote the paper.

ACKNOWLEDGMENTS

This work was supported by the Medical Research Council (MR/K011049/1 and MRC G0701140) and BBSRC (BB/K008773/1). The authors would like to thank Diamond Light Source for beamtime (proposal MX8997), and the staff of beamlines I02, I03, I04, and I24 for their assistance. In addition we extend our thanks to the staff of the Biomolecular Analysis Facility of the Faculty of Biology, Medicine and Health, University of Manchester, for assistance throughout the project.

Received: December 7, 2016
Revised: March 3, 2017
Accepted: May 10, 2017
Published: June 8, 2017

REFERENCES

Adams, P.D., Afonine, P.V., Bunkoczi, G., Chen, V.B., Davis, I.W., Echols, N., Headd, J.J., Hung, L.W., Kapral, G.J., Grosse-Kunstleve, R.W., et al. (2010). PHENIX: a comprehensive Python-based system for macromolecular structure solution. Acta Crystallogr. D Biol. Crystallogr. *66*, 213–221.

Ali, N., Zhang, L., Taylor, S., Mironov, A., Urbe, S., and Woodman, P. (2013). Recruitment of UBPY and ESCRT exchange drive HD-PTP-dependent sorting of EGFR to the MVB. Curr. Biol. *23*, 453–461.

Chen, Y.G. (2009). Endocytic regulation of TGF-beta signaling. Cell Res. *19*, 58–70.

Chen, Y.G., Wang, Z., Ma, J., Zhang, L., and Lu, Z. (2007). Endofin, a FYVE domain protein, interacts with Smad4 and facilitates transforming growth factor-beta signaling. J. Biol. Chem. *282*, 9688–9695.

Chen, V.B., Arendall, W.B., 3rd, Headd, J.J., Keedy, D.A., Immormino, R.M., Kapral, G.J., Murray, L.W., Richardson, J.S., and Richardson, D.C. (2010). MolProbity: all-atom structure validation for macromolecular crystallography. Acta Crystallogr. D Biol. Crystallogr. *66*, 12–21.

Chen, D.Y., Li, M.Y., Wu, S.Y., Lin, Y.L., Tsai, S.P., Lai, P.L., Lin, Y.T., Kuo, J.C., Meng, T.C., and Chen, G.C. (2012). The Bro1-domain-containing protein Myopic/HDPTP coordinates with Rab4 to regulate cell adhesion and migration. J. Cell Sci. *125*, 4841–4852.

Di Guglielmo, G.M., Le Roy, C., Goodfellow, A.F., and Wrana, J.L. (2003). Distinct endocytic pathways regulate TGF-beta receptor signalling and turnover. Nat. Cell Biol. *5*, 410–421.

Doyotte, A., Mironov, A., McKenzie, E., and Woodman, P. (2008). The Bro1-related protein HD-PTP/PTPN23 is required for endosomal cargo sorting and multivesicular body morphogenesis. Proc. Natl. Acad. Sci. USA *105*, 6308–6313.

Eden, E.R., Burgoyne, T., Edgar, J.R., Sorkin, A., and Futter, C.E. (2012). The relationship between ER-multivesicular body membrane contacts and the ESCRT machinery. Biochem. Soc. Trans. *40*, 464–468.

Ehrlich, M. (2016). Endocytosis and trafficking of BMP receptors: regulatory mechanisms for fine-tuning the signaling response in different cellular contexts. Cytokine Growth Factor Rev. *27*, 35–42.

Emsley, P., Lohkamp, B., Scott, W.G., and Cowtan, K. (2010). Features and development of Coot. Acta Crystallogr. D Biol. Crystallogr. *66*, 486–501.

Felberbaum-Corti, M., Van Der Goot, F.G., and Gruenberg, J. (2003). Sliding doors: clathrin-coated pits or caveolae? Nat. Cell Biol. *5*, 382–384.

Gahloth, D., Levy, C., Heaven, G., Stefani, F., Wunderley, L., Mould, P., Cliff, M.J., Bella, J., Fielding, A.J., Woodman, P., et al. (2016). Structural basis for selective interaction between the ESCRT regulator HD-PTP and UBAP1. Structure *24*, 2115–2126.

Goh, J.B., Wallace, D.F., Hong, W., and Subramaniam, V.N. (2015). Endofin, a novel BMP-SMAD regulator of the iron-regulatory hormone, hepcidin. Sci. Rep. *5*, 13986.

Hata, A., and Chen, Y.G. (2016). TGF-beta signaling from receptors to smads. Cold Spring Harb. Perspect. Biol. *8*, http://dx.doi.org/10.1101/cshperspect.a022061.

Hayes, S., Chawla, A., and Corvera, S. (2002). TGF beta receptor internalization into EEA1-enriched early endosomes: role in signaling to Smad2. J. Cell Biol. *158*, 1239–1249.

Heldin, C.H., and Moustakas, A. (2016). Signaling receptors for TGF-beta family members. Cold Spring Harb. Perspect. Biol. *8*, http://dx.doi.org/10.1101/cshperspect.a022053.

Hurley, J.H. (2015). ESCRTs are everywhere. EMBO J. *34*, 2398–2407.

Husebye, H., Halaas, O., Stenmark, H., Tunheim, G., Sandanger, O., Bogen, B., Brech, A., Latz, E., and Espevik, T. (2006). Endocytic pathways regulate Toll-like receptor 4 signaling and link innate and adaptive immunity. EMBO J. *25*, 683–692.

Ichioka, F., Takaya, E., Suzuki, H., Kajigaya, S., Buchman, V.L., Shibata, H., and Maki, M. (2007). HD-PTP and Alix share some membrane-traffic related proteins that interact with their Bro1 domains or proline-rich regions. Arch. Biochem. Biophys. *457*, 142–149.

Ichioka, F., Kobayashi, R., Katoh, K., Shibata, H., and Maki, M. (2008). Brox, a novel farnesylated Bro1 domain-containing protein that associates with charged multivesicular body protein 4 (CHMP4). FEBS J. *275*, 682–692.

Imamura, T., Oshima, Y., and Hikita, A. (2013). Regulation of TGF-beta family signalling by ubiquitination and deubiquitination. J. Biochem. *154*, 481–489.

Joosten, R.P., Joosten, K., Murshudov, G.N., and Perrakis, A. (2012). PDB_REDO: constructive validation, more than just looking for errors. Acta Crystallogr. D Biol. Crystallogr. *68*, 484–496.

Kabsch, W. (2010). XDS. Acta Crystallogr. D Biol. Crystallogr. *66*, 125–132.

Kavsak, P., Rasmussen, R.K., Causing, C.G., Bonni, S., Zhu, H., Thomsen, G.H., and Wrana, J.L. (2000). Smad7 binds to Smurf2 to form an E3 ubiquitin ligase that targets the TGF beta receptor for degradation. Mol. Cell *6*, 1365–1375.

Kharitidi, D., Apaja, P.M., Manteghi, S., Suzuki, K., Malitskaya, E., Roldan, A., Gingras, M.C., Takagi, J., Lukacs, G.L., and Pause, A. (2015). Interplay of endosomal pH and ligand occupancy in integrin alpha5beta1 ubiquitination, endocytic sorting, and cell migration. Cell Rep. *13*, 599–609.

Kim, J., Sitaraman, S., Hierro, A., Beach, B.M., Odorizzi, G., and Hurley, J.H. (2005). Structural basis for endosomal targeting by the Bro1 domain. Dev. Cell *8*, 937–947.

Lampugnani, M.G., Orsenigo, F., Gagliani, M.C., Tacchetti, C., and Dejana, E. (2006). Vascular endothelial cadherin controls VEGFR-2 internalization and signaling from intracellular compartments. J. Cell Biol. *174*, 593–604.

Lee, S., Joshi, A., Nagashima, K., Freed, E.O., and Hurley, J.H. (2007). Structural basis for viral late-domain binding to Alix. Nat. Struct. Mol. Biol. *14*, 194–199.

Lee, J., Oh, K.J., Lee, D., Kim, B.Y., Choi, J.S., Ku, B., and Kim, S.J. (2016). Structural study of the HD-PTP Bro1 domain in a complex with the core region of STAM2, a subunit of ESCRT-0. PLoS One *11*, e0149113.

Lobert, V.H., Brech, A., Pedersen, N.M., Wesche, J., Oppelt, A., Malerod, L., and Stenmark, H. (2010). Ubiquitination of alpha 5 beta 1 integrin controls fibroblast migration through lysosomal degradation of fibronectin-integrin complexes. Dev. Cell *19*, 148–159.

Ma, H., Wardega, P., Mazaud, D., Klosowska-Wardega, A., Jurek, A., Engstrom, U., Lennartsson, J., and Heldin, C.H. (2015). Histidine-domain-containing protein tyrosine phosphatase regulates platelet-derived growth factor receptor intracellular sorting and degradation. Cell Signal. *27*, 2209–2219.

Manteghi, S., Gingras, M.C., Kharitidi, D., Galarneau, L., Marques, M., Yan, M., Cencic, R., Robert, F., Paquet, M., Witcher, M., et al. (2016). Haploinsufficiency of the ESCRT component HD-PTP predisposes to cancer. Cell Rep. *15*, 1893–1900.

Marchese, A., Raiborg, C., Santini, F., Keen, J.H., Stenmark, H., and Benovic, J.L. (2003). The E3 ubiquitin ligase AIP4 mediates ubiquitination and sorting of the G protein-coupled receptor CXCR4. Dev. Cell *5*, 709–722.

McCullough, J., Fisher, R.D., Whitby, F.G., Sundquist, W.I., and Hill, C.P. (2008). ALIX-CHMP4 interactions in the human ESCRT pathway. Proc. Natl. Acad. Sci. USA *105*, 7687–7691.

Mellman, I., and Yarden, Y. (2013). Endocytosis and cancer. Cold Spring Harb. Perspect. Biol. *5*, a016949.

Mu, R., Dussupt, V., Jiang, J., Sette, P., Rudd, V., Chuenchor, W., Bello, N.F., Bouamr, F., and Xiao, T.S. (2012). Two distinct binding modes define the interaction of Brox with the C-terminal tails of CHMP5 and CHMP4B. Structure *20*, 887–898.

Olmos, Y., and Carlton, J.G. (2016). The ESCRT machinery: new roles at new holes. Curr. Opin. Cell Biol. *38*, 1–11.

Palacios, F., Tushir, J.S., Fujita, Y., and D'Souza-Schorey, C. (2005). Lysosomal targeting of E-cadherin: a unique mechanism for the down-regulation of cell-cell adhesion during epithelial to mesenchymal transitions. Mol. Cell Biol. *25*, 389–402.

Panopoulou, E., Gillooly, D.J., Wrana, J.L., Zerial, M., Stenmark, H., Murphy, C., and Fotsis, T. (2002). Early endosomal regulation of Smad-dependent signaling in endothelial cells. J. Biol. Chem. *277*, 18046–18052.

Parkinson, M.D., Piper, S.C., Bright, N.A., Evans, J.L., Boname, J.M., Bowers, K., Lehner, P.J., and Luzio, J.P. (2015). A non-canonical ESCRT pathway, including histidine domain phosphotyrosine phosphatase (HD-PTP), is used for down-regulation of virally ubiquitinated MHC class I. Biochem. J. *471*, 79–88.

Schoneberg, J., Lee, I.H., Iwasa, J.H., and Hurley, J.H. (2016). Reverse-topology membrane scission by the ESCRT proteins. Nat. Rev. Mol. Cell Biol. *18*, 5–17.

Schuh, A.L., and Audhya, A. (2014). The ESCRT machinery: from the plasma membrane to endosomes and back again. Crit. Rev. Biochem. Mol. Biol. *49*, 242–261.

Seet, L.F., and Hong, W. (2001). Endofin, an endosomal FYVE domain protein. J. Biol. Chem. *276*, 42445–42454.

Shi, Y., and Massague, J. (2003). Mechanisms of TGF-beta signaling from cell membrane to the nucleus. Cell *113*, 685–700.

Shi, W., Chang, C., Nie, S., Xie, S., Wan, M., and Cao, X. (2007). Endofin acts as a Smad anchor for receptor activation in BMP signaling. J. Cell Sci. *120*, 1216–1224.

Stefani, F., Zhang, L., Taylor, S., Donovan, J., Rollinson, S., Doyotte, A., Brownhill, K., Bennion, J., Pickering-Brown, S., and Woodman, P. (2011). UBAP1 is a component of an endosome-specific ESCRT-I complex that is essential for MVB sorting. Curr. Biol. *21*, 1245–1250.

Tsukazaki, T., Chiang, T.A., Davison, A.F., Attisano, L., and Wrana, J.L. (1998). SARA, a FYVE domain protein that recruits Smad2 to the TGFbeta receptor. Cell *95*, 779–791.

Vaccari, T., and Bilder, D. (2005). The Drosophila tumor suppressor vps25 prevents nonautonomous overproliferation by regulating notch trafficking. Dev. Cell *9*, 687–698.

von Zastrow, M., and Sorkin, A. (2007). Signaling on the endocytic pathway. Curr. Opin. Cell Biol. *19*, 436–445.

Zuo, W., Huang, F., Chiang, Y.J., Li, M., Du, J., Ding, Y., Zhang, T., Lee, H.W., Jeong, L.S., Chen, Y., et al. (2013). c-Cbl-mediated neddylation antagonizes ubiquitination and degradation of the TGF-beta type II receptor. Mol. Cell *49*, 499–510.

STAR★METHODS

KEY RESOURCES TABLE

REGENTS or RESOURCES	SOURCE	IDENTIFIER
Antibodies		
anti-EEA1 (Mouse)	BD Biosciences	RRID: AB_397830
anti-HA (Mouse)	Santa Cruz	RRID: AB_627809
anti-Myc 9B11 Clone (Mouse)	Cell Signalling Technology	RRID: AB_331783
anti-His (Mouse)	Sigma	RRID: AB_260015
anti-HA (Rat)	Roche	RRID: AB_390919
anti-HD-PTP (Rabbit)	Proteintech	RRID: AB_2173382
Cy3 AffiniPure Donkey Anti-Rat IgG (H+L)	Jackson ImmunoResearch Laboratories (PA, USA)	RRID: AB_2340667
Alexa Fluor® 488 AffiniPure Donkey Anti-Mouse IgG (H+L)	Jackson ImmunoResearch Laboratories (PA, USA)	RRID: AB_2340846
anti-SARA	Proteintech	RRID: AB_11182938
anti-Endofin	Proteintech	RRID: AB_2288795
Peptides		
Synthetic Peptides (CHMP4s, SARA & Endofin)	Generon Ltd, UK	NA
Commercial Assays		
pEIMAX	Polysciences	NA
“Matchmaker Gold” system	Clonetech	Cat#630489
Deposited Data		
PDB accession code	www.rcsb.org	5MK0, 5MK1, 5MK2, 5MK3, 5MJY, 5MJZ
Experimental Models: Cell Lines		
HeLaM	Laboratory of Margaret Robinson, Cambridge Institute for Mediacl Research, Cambridge. UK.	N/A
HEK93	ATCC	CRL-1573
Software and Algorithms		
ProteOn Manager software	Bio-Rad	NA
CCP4i		http://www.ccp4.ac.uk
Phenix		https://www.phenix-online.org
PDB-redo		https://pdb-redo.eu

CONTACT FOR REAGENT AND RESOURCE SHARING

Further information and requests for reagents should be directed to corresponding authors Philip Woodman (philip.woodman@manchester.ac.uk) and Lydia Tabernero (lydia.tabernero@manchester.ac.uk).

METHOD DETAILS

Expression Constructs for Heterologous Expression

HD-PTP$_{Bro1}$ (1-361) was cloned in a pNIC28-Bsa4 vector (Gift from Opher Gileadi (Addgene # 26103)) in its Ligase Independent Cloning (LIC) site after generating sticky ends with T4 DNA Polymerase (NEB). HD-PTP$_{Bro1-CC}$ (1-714) and Alix$_{Bro1}$ (1-359) were cloned in a pET28a vector between the Nde1 and Xho1 cleavage sites. CHMP4B (1-224) and CHMP4B ΔCt6 (1-118) were cloned in a pGEX-4T1 vector between the EcoRI and XhoI sites. Point mutations were introduced by quick-change primers using Phusion DNA polymerase enzyme (NEB). HA-tagged HD-PTP (wt and L202D, I206D mutants) have been described previously (Doyotte et al., 2008). For the yeast two-hybrid screening, HD-PTP$_{FL}$ was cloned into pGBT9, HD-PTP$_{Bro1-CC}$ and Alix$_{Bro1-V}$ cloned into pGBKT7, and CHMP4B cloned into in pACT2.2 as described (Stefani et al., 2011). Human endofin and human SARA were cloned into pGADT7 using the In-Fusion cloning system (Clontech). Endofin and SARA with C-terminal myc tags (endofin-myc, SARA-myc) were generated by

subcloning into pcDNA5 (Invitrogen). FL endofin-Strep-tag and endofin(1-84)-Strep-tag were generated by subcloning into pTriEx-5 (Novagen) modified to generate C-terminal Strep-tags. The sequences of all constructs were confirmed by DNA sequencing. HD-PTP$_{Bro1}$, HD-PTP$_{Bro1-CC}$ and Alix$_{Bro1}$ constructs were transformed in *E.coli* BL21 (DE3) cells and GST-CHMP4B and GST-CHMP4BΔCT in C41 (DE3) cells. Cells containing plasmids were grown in LB media in the presence of either 50 μg/ml Kanamycin (HD-PTP$_{Bro1}$, HD-PTP$_{Bro1-CC}$ and Alix$_{Bro1}$) or 100 μg/ml Ampicillin (GST-CHMP4B and GST-CHMP4BΔCT) up to an O.D$_{600}$ of 0.6-0.8 at 37°C. Over-expression of proteins was induced overnight at 20°C with 0.1 mM IPTG.

Protein Purification

HD-PTP$_{Bro1}$, HD-PTP$_{Bro1-CC}$ and Alix$_{Bro1}$ were expressed as N-terminal His$_6$-tagged proteins. Bacterial cell pellets were re-suspended in lysis buffer containing 20 mM HEPES pH 7.4, 0.5 M NaCl, 0.1% Triton-X 100, 2 mM PMSF, 10 mM Imidazole and cells were disrupted by sonication. The lysate was cleared by centrifugation at 12,400 g for 1h. His-tagged proteins and then purified using Nickel-beads (Qiagen) pre-equilibrated in 20 mM HEPES pH 7.4, 0.5 M NaCl, 10 mM Imidazole. Proteins were eluted with 20 mM HEPES pH 7.4, 0.5 M NaCl, 250 mM Imidazole. All the protein-containing fractions were buffer exchanged with 10-kDa Vivaspin concentrators (Sartorius) and further purified using a MonoQ column (GE Healthcare) pre-equilibrated in 20 mM Tris-Cl pH 8.0 2 mM EDTA, 2 mM DTT and eluted with a linear gradient of NaCl. Final purification was performed using gel-filtration on a Superdex 200 column (GE Healthcare) in 20 mM HEPES pH 8.0, 150 mM NaCl. For GST-CHMP4B and GST-CHMP4BΔCT, cells were re-suspended in 50 mM Tris-Cl pH 8.0, 0.25 M NaCl, 2mM DTT, 0.1% Triton-X-100, 2 mM PMSF lysis buffer. Cells were lyzed by sonication and the supernatant was cleared by centrifugation at 12,400 g for 1h. Supernatant was loaded on a 5 ml GSTrap HP column (GE Healthcare) pre-equilibrated in binding buffer (50 mM Tris-Cl pH 8.0, 0.25 M NaCl, 2 mM DTT) and eluted with binding buffer containing 20 mM reduced glutathione. Fractions containing GST-CHMP4B and GST-CHMP4BΔCT were pooled, concentrated and buffer exchanged in 50 mM Tris-Cl pH 8.0, 0.25 M NaCl, 2 mM DTT.

Biosensor Binding Studies

Biosensor based binding experiments were performed using a ProteOn XPR36 surface Plasmon resonance instrument (Bio-Rad Laboratories). The ProteOn XPR36 is multiplex system that can be used to provide simultaneous flow of up to six analyte channels (A1-A6) over up to six ligand channels (L1-L6). The running buffer used was 10 mM HEPES pH 7.4, 150 mM NaCl, 0.05% Tween 20. Immobilization of His$_6$-tagged HD-PTP and a negative control protein, His$_6$-tagged MptpB, was performed on a HTE chip (Bio-Rad Laboratories) in the vertical orientation. Proteins were diluted in running buffer to a final concentration of 50-100 μg/ml and 150 μl was injected at a flow rate of 30 μl/min The immobilization level of proteins was typically 5000-8000 response unit (RU). All experiments were performed at 25°C. Synthetic peptides spanning the binding regions of CHMP4 A, B and C, and SARA and endofin, were obtained from Generon Ltd, UK. For equilibrium binding measurements these peptides were used as analytes. Peptide stocks were prepared in running buffer just prior the binding experiments. Peptides were injected (50 μl at 100 μl/min) in the horizontal orientation using five serially diluted peptide concentrations (channels A1-A5) alongside a buffer control (channel A6). Peptide concentrations were chosen to give a suitable spread of responses below and above half-maximal binding.

Crystallization

For crystallization, the N-terminal His$_6$-tag was cleaved from HD-PTP$_{Bro1}$ (1-361) with TEV protease, incubating overnight at 4°C followed by Ni-affinity chromatography. The digested protein was further purified as described above with MonoQ and gel filtration and left in the gel filtration buffer (20 mM HEPES pH 7.4, 0.15 M NaCl, 2 mM EDTA, 2 mM DTT) prior to concentration. Apo-HD-PTP$_{Bro1}$ crystals were obtained in 0.2 M L-Na-Glutamate, 0.2 M Alanine, 0.2 M Glycine, 0.2 M Lysine HCl, 0.2 M Serine, 0.1 M Imidazole; MES monohydrate at pH 6.5 and 30% Ethylene glycol/PEG8K. For the co-crystallization experiments HD-PTP$_{Bro1}$ (1.5 mg/ml) was mixed with the CHMP4 and SARA/endofin peptides to a 1 mM final concentration of each peptide and incubated overnight at 4°C. The protein-peptide mixture was concentrated up to 11 mg/ml (HD-PTP$_{Bro1}$ concentration) and used for the crystallization screenings. The best diffracting crystals of HD-PTP$_{Bro1}$ with CHMP4A were obtained in 0.1 M Tris pH 7.8, 5% γ-PGA-LM (poly-γ-glutamic acid low molecular weight polymer), 20% PEG3350. HD-PTP$_{Bro1}$ co-crystals with CHMP4B were obtained in 0.2 M $CaCl_2$, 0.1 M MES pH 6.0, 20% PEG 6K, and HD-PTP$_{Bro1}$ co-crystals with CHMP4C were obtained in 0.2 M Potassium thiocyanate, 0.1 M Bis-Tris propane pH 6.5, 20% PEG 3350. Co-crystals of HD-PTP$_{Bro1}$ with endofin and SARA were grown in 0.2 M ammonium tartrate dibasic, 20% PEG3350 and 0.1 M MMT buffer pH 9.0, 25% PEG1500 respectively. All crystals appeared overnight at 21°C.

Data Collection and Structure Determination

Crystals were cryo-protected in mother liquor supplemented with 10-15% PEG200 and flash frozen in liquid nitrogen. All data were collected at Diamond beam-lines (i02, i03, i04 & i24) at 100K. Data were indexed and integrated in XDS (Kabsch, 2010) and the structures were solved by Molecular Replacement as implemented in PHENIX Phaser-MR (Adams et al., 2010) using the HD-PTP Bro1 domain (3RAU.pdb) as the search model. Following Molecular Replacement the initial structures were automatically built using PHENIX Autobuild and completed through an iterative cycle of manual building and refinement in Coot (Emsley et al., 2010) and Phenix.refine (Adams et al., 2010). Validation of the structures with both Molprobity (Chen et al., 2010) and PDB_REDO (Joosten et al., 2012) was integrated into the iterative rebuild and refinement process. Final statistics of the six refined structures are presented in Table 1. All the molecular structure images were prepared using the PyMOL (Schrödinger, LLC).

Antibodies

The following commercial antibodies were used: Mouse: anti-EEA1 (BD Biosciences Cat. No. 610457); anti-HA (for immunoblotting) (Santa Cruz Cat. No Sc-7392); anti-myc 9B11 clone (Cell Signalling Technology Cat. No. 2276); anti-His (Sigma Clone His-1; Cat. No. H1029). Rat: anti-HA (for IF) (Roche; clone 3F10). Rabbit: anti-HD-PTP (Proteintech Cat. No.102472-1-AP), anti-endofin (Proteintech Cat. No. 13118-2-AP), anti-SARA (Proteintech Cat. No. 22033-1-AP). The anti-endofin and anti-SARA antibodies were verified by siRNA (Figure S1A) using OnTargetPlus siRNA Smartpool reagents (Dharmacon) for endofin (LQ-020254-01-0005) and SARA (LQ-011939-00-0005), vs a Dharmacon control siRNA. Fluorescent secondary antibodies for IF or for immunoblotting were from Jackson ImmunoResearch Laboratories (PA, USA). Rat-Mouse double labeling IF experiments used Cy3 AffiniPure Donkey Anti-Rat IgG (H+L), immunoadsorbed to prevent cross-reactivity towards mouse IgG (Catalogue 712-165-153), and Alexa488 AffiniPure Donkey Anti-Mouse IgG (H+L), immunoadsorbed to prevent cross-reactivity towards rat IgG (catalogue 715-545-150) (Jackson ImmunoResearch laboratories).

Yeast Two Hybrid Analysis

Yeast two-hybrid screening was performed by Hybrigenics, S.A., Paris, France (http://www.hybrigenics-services.com). The coding sequence for amino acids 1-714 of the human HD-PTP/PTPN23 protein (GenBank accession number gi: 110681717) was PCR-amplified and cloned into pB27 as a C-terminal fusion to LexA (N-LexA-HD-PTP-C). The construct was checked by sequencing and used as a bait to screen a random-primed human placenta cDNA library constructed into pP6 as previously described (Stefani et al., 2011). See also Table S1. His+ colonies were selected on a medium lacking tryptophan, leucine and histidine. The prey fragments of the positive clones were amplified by PCR and sequenced at their 5' and 3' junctions. Interactions were further tested by directed yeast two-hybrid, using the Clontech "Matchmaker Gold" system (Clontech) as described previously (Stefani et al., 2011). The following modifications were made to allow yeast growth and expression of all constructs, including full length HD-PTP and CHMP4B, which grew slowly under standard conditions. Transformants into the Y2H Gold strain of yeast were grown on DDO plates (deficient in tryptophan and leucine to select for transformants) with 0.4% D+ Glucose and 1% D+ Galactose. Transformant colonies were inoculated into 2.5 ml liquid DDO media in triplicate (per condition) in 2% D+ Glucose and grown for 24-48 hours. A 10μl inoculating loop was used to transfer some of each liquid culture to a square on parallel DDO (to select for transformants) and QDO (additionally deficient in histidine and adenine to select for interactions) plates, with 0.4% D+ Glucose and 1% D+ Galactose. Figures show QDO plates only. Colonies grew on parallel DDO plates for all combinations displayed, confirming the expression of both constructs in all experiments. At least 3 independent replicates were performed for each experiment, with each replicate containing 3 technical repeats.

Cell Culture, Transfections and Microscopy

Cells were cultured in DMEM containing 10% FBS, non-essential amino acids and 1% Penicillin-Streptomycin. HeLaM cells were used for fluorescence studies, and HEK293 cells used for most biochemical studies. Transfections were performed using pEIMAX (Polysciences, Warrington, PA). For the HD-PTP-myc/endofin-Strep IP in Figure S1B, HeLa cells stably expressing HD-PTP-myc were induced with doxycyclin as described (Ali et al., 2013). siRNA transfections were performed with siRNA Smartpool reagents to endofin and SARA, or control siRNA, using INTERFERin (Polyplus Transfection). Cells were transfected for 20 nM siRNA over 72 hr. For immunofluorescence experiments, cells were fixed in 3% formaldehyde and quenched with glycine, then permeabilized for 3 min in PBS containing 0.1% Triton X-100. Fluorescence was imaged on an Olympus BX60 upright microscope fitted with a 60 x 1.4 NA Plan Apo objective and CoolSnap ES camera, and 12-bit images captured using MetaVue software. All images were opened as 16-bit grey-scale images and scaled using linear transformations in ImageJ, then converted to 24-bit RGB files in PhotoShop CS. At least 3 independent replicates were performed for each experiment unless otherwise stated. For scoring, 3 random images were selected from each sample and 15-20 cells in total from each experiment scored for the presence of HA-HD-PTP in clusters that also labelled for endofin-myc or SARA-myc. Note that in some cells expressing low levels of HA-HD-PTP alone, faint HA-HD-PTP staining could be detected on membrane structures, though these were not clustered. However, cytoplasmic staining of HD-PTP dominated in most cells.

Immunoprecipitations from Cell Lysates

For native immunoprecipitations, cells were lyzed in immunoprecipitation buffer (IP1 buffer: 25 mM Tris-HCl pH 7.5, 40 mM NaCl, 0.5% IGEPAL) and supplemented with protease inhibitor cocktail (PIC III, Sigma). Lysates were centrifuged at 14,000 rpm for 15 min at 4°C and the supernatants incubated with the indicated antibodies at 4°C. Following overnight incubation, immune complexes were captured by incubating with anti-HA-sepharose (Sigma), pre-blocked with 50 mg/ml BSA in IP1 buffer, or control beads. Alternatively, IP samples were precipitated using protein A-sepharose (Invitrogen), pre-blocked as above, with non-immune IgG used as control. The beads were then washed four times in IP1 buffer and protein complexes were eluted with SDS PAGE buffer. All control IPs were performed using non-immune sera or IgGs. Immunoblotting was performed using PVDF membrane (Millipore). Blots were imaged using a Odyssey Sa imager and quantified using Image Studio (LI-COR Biosciences). At least 3 independent replicates were performed for each experiment.

In Vitro Translation and Binding Assays

Endofin(1-84) constructs encoded on a pTriex5 vector modified to generate a C-terminal Strep-tag were amplified using Pwo Polymerase (Roche). RNA was synthesized from the PCR product using T7 RNA polymerase (Promega) and protein was translated in nuclease treated rabbit reticulocyte lysate (Promega) containing ^{35}S-methionine (Perkin Elmer), and 100 units of RNasin (Promega) for 1h at 30°C. Samples were incubated at 30°C with 1 mM puromycin for a further 10 min. For binding experiments, 20 μl translated protein was incubated with 5 μg His_6-HD-$PTP_{Bro1-CC}$ in 250 μl IP2 buffer (20mM Hepes, pH7.4, 100mM NaCl, 1mM $MgCl_2$, 1% (w/v) Triton X-100) for 2h at 4°C, then overnight with 3 μl anti-His antibody. Samples were incubated with 20μl protein A-sepharose beads (Invitrogen) for 2h at 4°C, then washed 3 x in IP2 buffer. Following SDS-PAGE gels were dried and visualized using phosphorimaging and quantified using AIDA (Raytest). For CHMP4B binding experiments, full length or truncated GST-CHMP4B (50 μg/ml) and His_6-HD-$PTP_{Bro1-CC}$ (5 μg) were incubated in 200 μl IP2 buffer containing 250 mM NaCl for 2h at 4°C prior to immunoprecipitation with anti-His. For competition experiments endofin 22-mer peptide was added at a final concentration of 20 μM. At least 3 independent replicates were performed for all experiments.

QUANTIFICATION AND STATISTICAL ANALYSIS

Biosensor Binding Studies

All the binding sensograms were collected, processed and analyzed using the integrated ProteOn Manager software (Bio-Rad Laboratories), using the equilibrium binding mode: Response = $[A] * R_{max}/([A] + K_D)$, where [A] is the analyte concentration and R_{max} is the maximum response. At least 3 independent replicates were performed for each experiment. Values in the text and figure legends indicate mean +/- SD (standard deviation).

Immunoprecipitations and Binding Assays

Quantitation was performed from at least three independent experiments. Values shown in text and figure legends are mean +/- SD (standard deviation) with n shown for each experiment. Statistics for immunoprecipitations were performed in Prism7 (GraphPad) using the unpaired t-test with Welch's correction: ***: $p<0.001$; **: $p<0.01$; *: $p<0.05$.

DATA AND SOFTWARE AVAILABILITY

Accession Numbers

The atomic coordinates for the structures presented in this study have been deposited in the Protein Data Bank under ID codes: 5MK0, 5MK1, 5MK2, 5MK3, 5MJY and 5MJZ.

Structure, Volume *25*

Supplemental Information

Structural Basis for Specific Interaction of TGFβ Signaling Regulators SARA/Endofin with HD-PTP

Deepankar Gahloth, Colin Levy, Louise Walker, Lydia Wunderley, A. Paul Mould, Sandra Taylor, Philip Woodman, and Lydia Tabernero

Supplementary Information

Supplementary Figures S1, S2, S3; Supplementary Table 1

Figure S1 (related to Figure 1 and Figure 2)

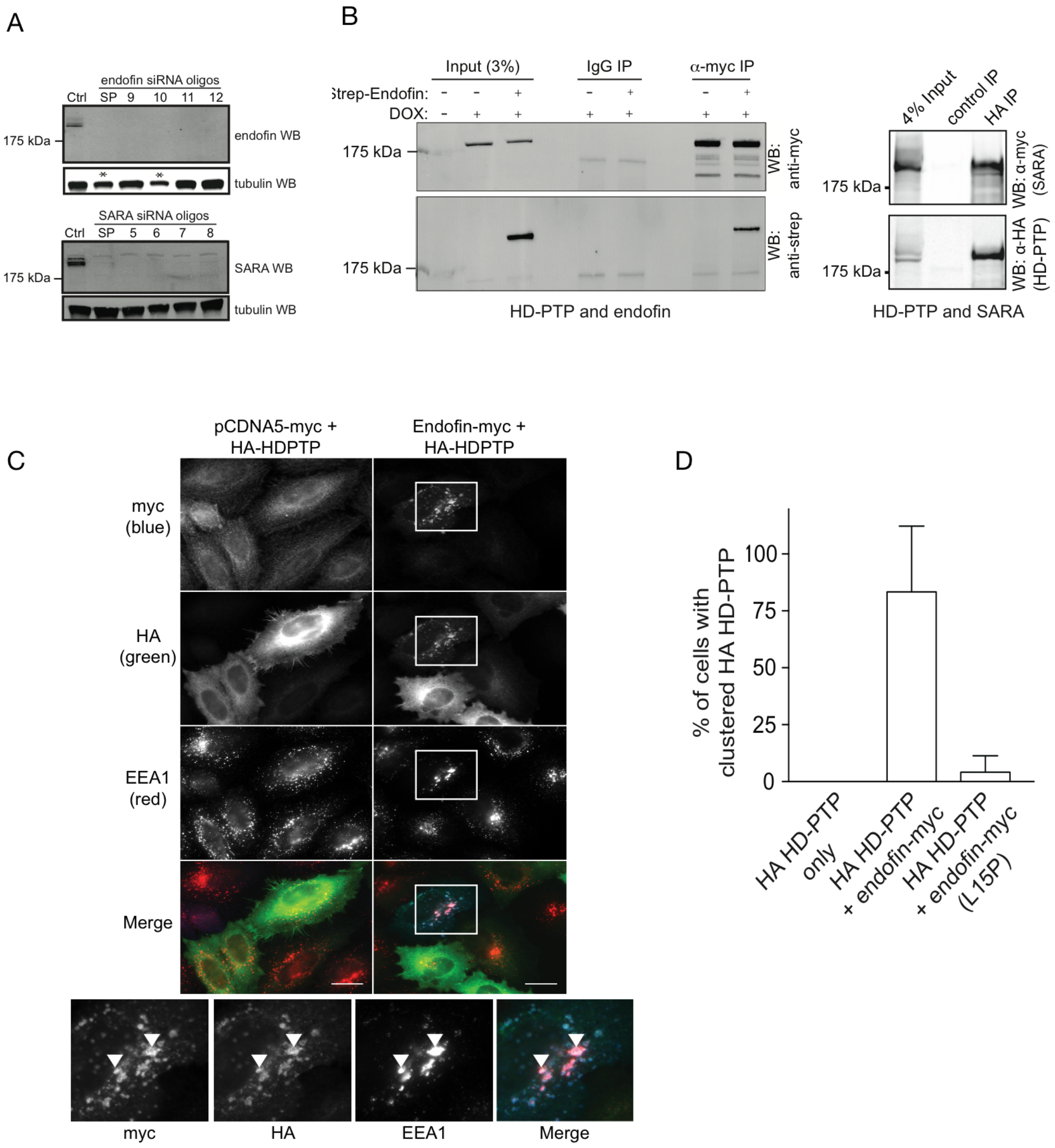

(A) HeLaM cells were transfected with siRNAs against endofin, SARA, or with a control siRNA and extracts were immunoblotted for endofin or SARA, with tubulin as a loading control. Note that endofin oligo 10 was mildly toxic (asterisks). (B) Left panel: HD-PTP-myc Flip-In HeLa cells were transiently

transfected with endofin-Strep-tag and induced with doxycycline (DOX). Control IgG or anti-myc IPs were blotted as indicated. Right panel: HEK293 cells co-expressing HA-HD-PTP and SARA-myc were subjected to control or anti-HA IPs and immunoblotted as indicated. (C) Cells were transfected with empty vector and HA-HD-PTP (left panels), or with endofin-myc and HA-HD-PTP (right panels), and stained with anti-myc (blue) and anti-HA (green), as well as with anti-EEA1 (red) to label early endosomes. Bars = 10 µm. Boxed regions are magnified x 3 and displayed below. Arrowheads indicate examples of co-localisation between HA-HD-PTP and endofin-myc occurring on early endosomes. (D) Cells were transfected with HA-HD-PTP and WT endofin-myc or endofin-myc^{L15P}. Colocalisation was scored in 3 independent experiments. Values are means +/- SD.

Figure S2 (related to figure 4)

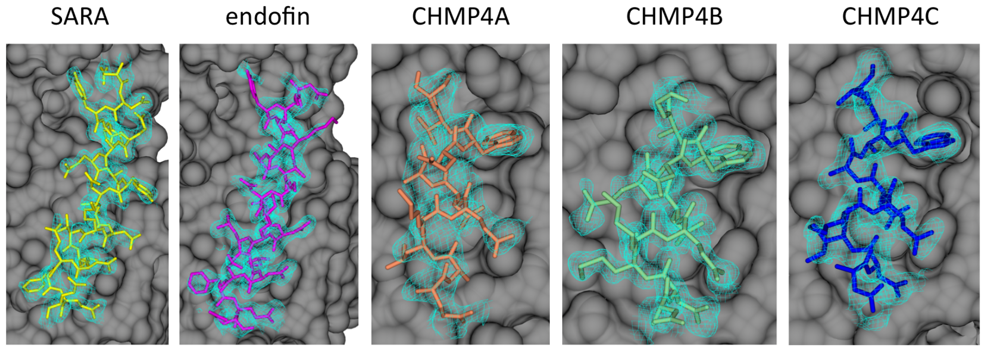

Electron density maps for the peptides in each structure presented in the study. Each of the five peptides (SARA - yellow, endofin - purple, CHMP4A - orange, CHMP4B - green & CHMP4C - blue) is shown in stick representation along with a surface rendering of their respective HD-PTP domains (grey). Fo-Fc omit electron density (cyan) contoured at 2 sigma is shown for each

peptide. Figure generated in CCP4MG v2.10.4 (McNicholas et al., 2011).

Figure S3 (related to Figure 7)

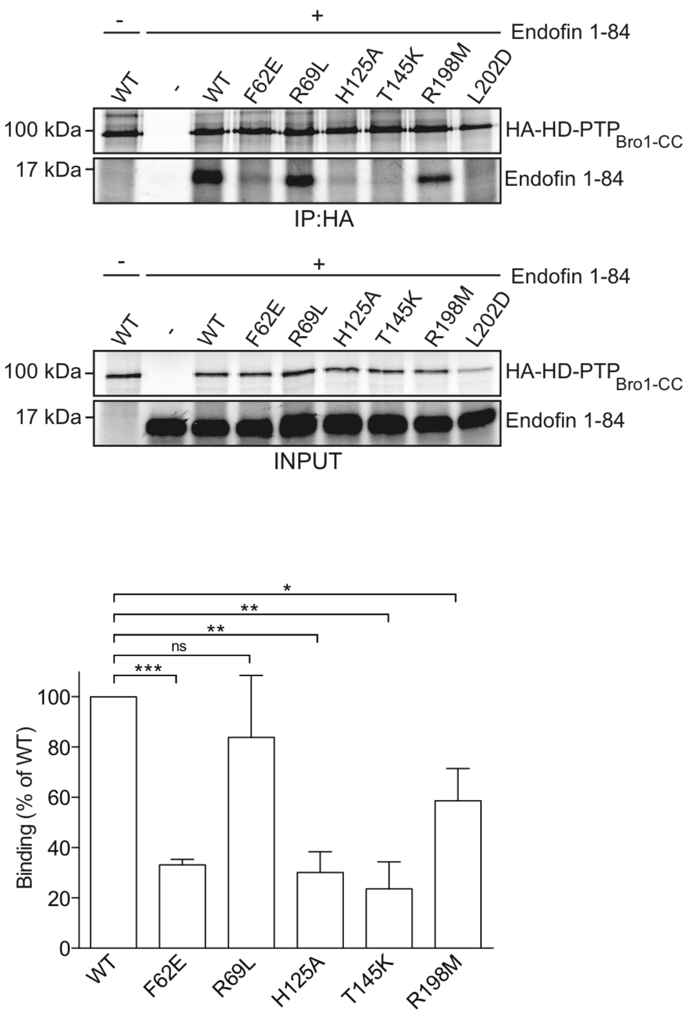

Top: wild type (WT) HA-HD-PTP$_{Bro1\text{-}CC}$ or the indicated mutants were co-translated with endofin1-84-myc in reticulocyte lysates. Translation products (input) are displayed in the lower panels. Samples were immunoprecipitated with anti-HA beads and examined by phosphorimaging to identify HA-HD-PTP$_{Bro1\text{-}CC}$ and co-immunoprecipitated endofin1-84-myc (upper panels). Bottom: data from 3 independent experiments +/- SD. Note that data for the L202D mutant were not included in the quantitation because this mutant

translated poorly compared to the other constructs. Mutation of HA-HD-$PTP_{Bro1\text{-}CC}$ residues F62, H125 and T145 at the S-site and of L202 at the common site impair binding of the endofin 1-84 fragment. Mutation of R69 or R198 had a milder effect with R69 not significantly different from WT. Statistics were performed using the unpaired t-test with Welch's correction: ***: $p<0.001$; **: $p<0.01$; *: $p<0.05$.

Table S1. HD-PTP interactors revealed in a Y2H screen (related to STAR Methods section).

$HD\text{-}PTP_{Bro1\text{-}CC}$ was used as bait to screen a placental cDNA library for interactors. The following ESCRT and endocytic proteins were identified (number of clones shown in brackets). Binding to UBAP1 and STAM2 have already been reported (Stefani et al., 2011, Ali et al., 2013).

ESCRTs/ESCRT-related	Endosomal
CHMP4B (1)	Endofin (5)
CHMP5 (1)	SARA (4)
STAM2 (5)	Rabep1 (7)
UBAP1 (5)	
SPG20 (3)	
